# Chromatin Ubiquitination Guides DNA Double Strand Break Signaling and Repair

**DOI:** 10.3389/fcell.2022.928113

**Published:** 2022-07-05

**Authors:** Ksenia G. Kolobynina, Alexander Rapp, M. Cristina Cardoso

**Affiliations:** Department of Biology, Cell Biology and Epigenetics, Technical University of Darmstadt, Darmstadt, Germany

**Keywords:** chromatin, DNA damage response, DNA repair, double-strand breaks, ubiquitination

## Abstract

Chromatin is the context for all DNA-based molecular processes taking place in the cell nucleus. The initial chromatin structure at the site of the DNA damage determines both, lesion generation and subsequent activation of the DNA damage response (DDR) pathway. In turn, proceeding DDR changes the chromatin at the damaged site and across large fractions of the genome. Ubiquitination, besides phosphorylation and methylation, was characterized as an important chromatin post-translational modification (PTM) occurring at the DNA damage site and persisting during the duration of the DDR. Ubiquitination appears to function as a highly versatile “signal-response” network involving several types of players performing various functions. Here we discuss how ubiquitin modifiers fine-tune the DNA damage recognition and response and how the interaction with other chromatin modifications ensures cell survival.

## Introduction

DNA damage is the major threat to genome stability, especially in the form of a double-strand break (DSB). Various exogenous and endogenous damaging agents can cause DSBs. Unrepaired DSBs are one of the most harmful damage types, causing cell cycle arrest, apoptosis, or malignant transformation (Bartkova et al., 2005; Gorgoulis et al., 2005; Negrini et al., 2010). To cope with the threat, DNA damage response (DDR) and repair systems have evolved. There are two major DSB repair pathways: non-homologous end joining (NHEJ) and homologous recombination (HR). The principal difference between them, which is also reflected in the fidelity of repair, is whether or not DSB ends are resected, i.e. endure 5′–3′ degradation of one strand at each side of the break. The NHEJ, or mammalian classical NHEJ (cNHEJ), is a very fast repair pathway that employs ligation of ends with minimum resection and sequence homology. In contrast, the multistep HR is driven by broken ends resection and homology search with the single-strand DNA using an intact copy of the damaged locus as a template for repair. The NHEJ is functional throughout the cell cycle while the HR is a preferable choice in the S-G2 stages of the cell cycle when a sister chromatid is available for template-guided repair. Naturally, DNA damage and the DDR both happen in the context of chromatin. Chromatin, comprising DNA together with associated histone and non-histone proteins, determines the structure and function of the genome. At the primary level, chromatin is constituted by approximately 147 base pairs of DNA wrapped twice around eight core histones (H2A, H2B, H3, H4) forming nucleosomes (Luger et al., 1997). Nucleosome arrays compact and fold further into high-order structures creating complex multilevel 3D chromatin architecture. With each new level of folding, more and more factors are involved in the decision process that regulates the chromatin structure. In addition, DNA and histones can be chemically modified. Histone post-translational modifications (PTM), such as phosphorylation, methylation, acetylation, ubiquitination as well as histone variants modulate histone dynamics and DNA association. Together with the linker histone H1 and many non-histone proteins these factors directly define the physical properties of the chromatin fiber including secondary structure, accessibility, phase separation properties, and mobility, hence ultimately affecting damage induction, recognition, and processing.

## Chromatin Remodeling and Modifications Associated With the DNA Double Strand Break Response

In an undamaged state, chromatin exists in a variety of functionally and topologically different domains, which can change dynamically. Based on the degree of compaction, originally based upon contrast staining with basic dyes (Heitz, 1928) chromatin is classified into more compacted, usually defined as transcriptionally inert heterochromatin and open, transcriptionally active, gene-rich euchromatin. Each of them is characterized by unique epigenetic and structural properties. Chromatin conformation reflects the role of the underlying sequences in the genome: centromeric and pericentromeric regions of chromosomes are organized into constitutive heterochromatin, which is believed to stay repressed among all cell types (Saksouk et al., 2015). On the other hand, facultative heterochromatin contains genes, which are silent in the corresponding cell type-dependent on the developmental state. In response to various stimuli, facultative heterochromatin is remodeled into euchromatin and vice versa, which ensures that the correct genes are expressed.

The pre-existing conformation of chromatin is a factor that affects DNA damage induction. Additional factors include the cell cycle stage (Ward and Chen, 2001), transcriptional activity (Falk et al., 2008; Herrera-Moyano et al., 2014; Prendergast et al., 2020; Bayona-Feliu and Aguilera, 2021), DNA secondary structure such as G-quadruplexes (Kumari et al., 2019), as well as the type of DNA damage and its source (e.g. LET (linear energy transfer) of ionizing radiation (Löbrich et al., 1996; Sutherland et al., 2001; Radulescu et al., 2004). When exposed to ionizing irradiation, the more compacted heterochromatin environment has been reported to physically shield DNA from damage and thus decrease the frequency and severity of the lesions formed (Elia and Bradley, 1992; Yoshikawa et al., 2008; Takata et al., 2013; Cannan et al., 2014; Brambilla et al., 2020). The number of water molecules available for radiolysis and subsequent DNA damage generation in condensed regions is considered to be lower than in decondensed ones. Along with this line, tightly packed nucleosomes in heterochromatin should show decreased accessibility for damage sensors and repair proteins, and consequently, decreased repair efficiency. Yet, this hypothesis of the impervious heterochromatin was questioned (Caridi et al., 2017). Chromatin compaction affects lesion induction, but compacted regions are not as irresponsive as was suggested before (Jakob et al., 2011; Chagin et al., 2019). The complex relationship between the initial chromatin structure and the DDR was summarized in two models. The “access-repair-restore” model came first and depicted chromatin as an obstacle for the repair machinery (Smerdon, 1991). Later this model was revised according to the evidence that chromatin actively participates in the DDR and provides the necessary context for repair. The updated model was named “prime-repair-restore”, where the prime step comprises both the contribution of the initial chromatin landscape and its remodeling to facilitate damage signaling (Soria et al., 2012). At the prime step, the PTMs on histone and non-histone proteins, corresponding to the pre-existing eu- or heterochromatin epigenetic landscape, change to the ones of the repair-associated epigenetic landscape, thus, promoting the formation of a 3D repair domain. Interaction of proteins and successful crosstalk between damage signaling and repair mechanism at the sites of DSBs is achieved by a highly controlled network of PTMs. The latter changes protein stability, charge, activity, structure, or interaction with other players of the pathway. Among the various PTMs of proteins at the sites of DNA lesions are phosphorylation, acetylation, methylation, poly(ADP-ribosyl)ation, ubiquitination, and sumoylation (Kouzarides, 2007). The *de novo* establishment of the PTMs is catalyzed by writer enzymes, with the removal of the PTMs being done by eraser enzymes and the transduction of the signal downstream in the cascade being carried out by reader proteins. The chromatin modifications and their role are, therefore, highly dynamic and tightly regulated at multiple levels.

**FIGURE 1:**
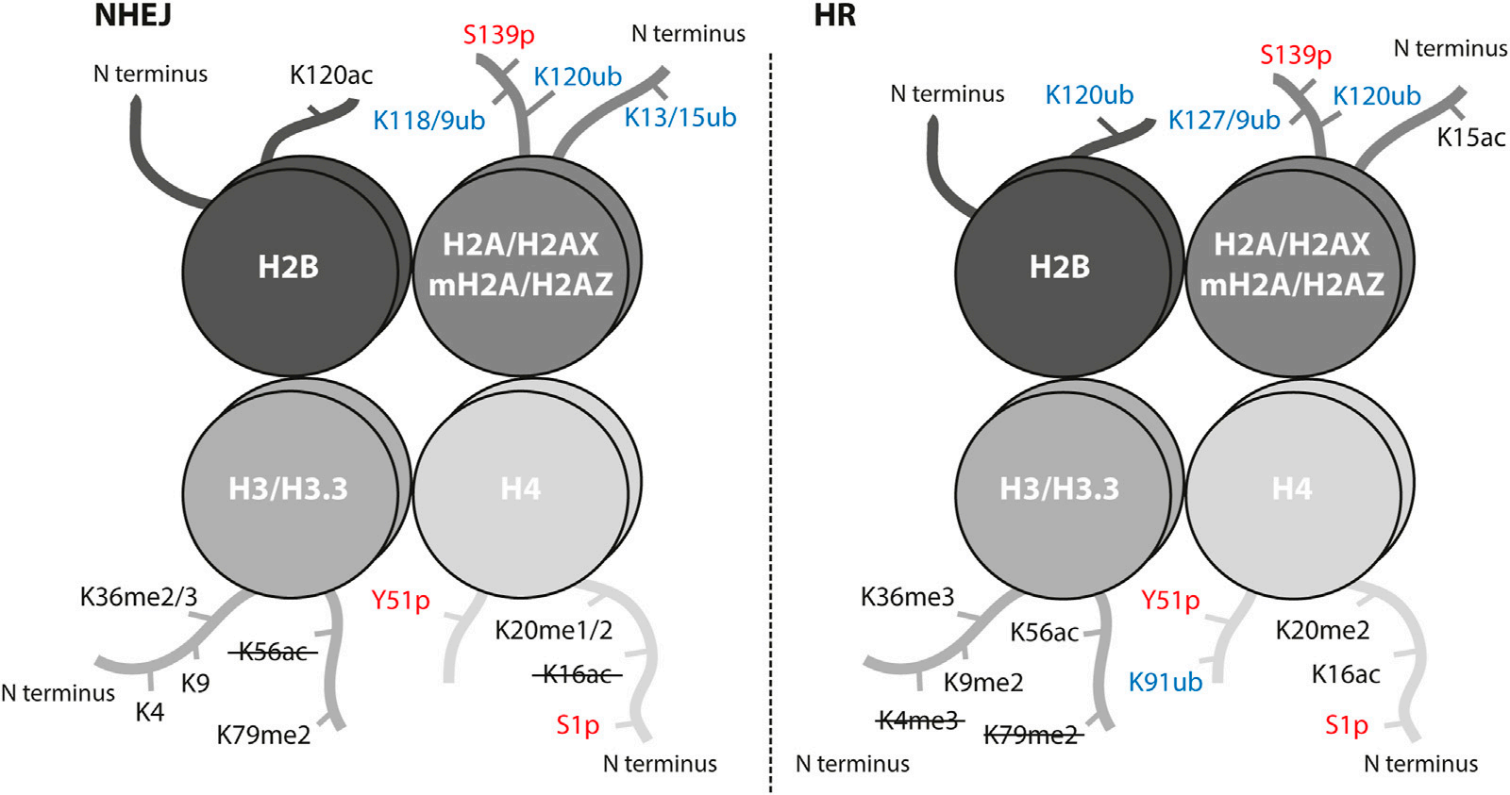
Histone post-translational modifications associated with NHEJ and HR repair pathways. Interaction of proteins and successful crosstalk between damage signaling and repair mechanism at the sites of DSBs is achieved by a highly controlled network of PTMs. Histone PTMs play a role in DSB repair pathway choice and serve as binding sites for repair proteins. Ubiquitination marks in blue, phosphorylation marks in red; ub, ubiquitination; p, phosphorylation; ac, acetylation; me, methylation. The crossed-out mark means it is removed in the repair pathway.

To maintain genome stability, two major DSB repair pathways (non-homologous end joining (NHEJ) and homologous recombination (HR)) have evolved. Both pathways are characterized by a different chromatin landscape (Figure 1).

The key event in DSB repair is the recognition of DSB by the MRN complex (MRE11-RAD50-NBS1) and phosphorylation of serine 139 (termed γH2AX) on the histone variant H2AX by the PI3 kinase-related protein kinases ATM, ATR and DNA-PKcs (Rogakou et al., 1998; Sedelnikova et al., 2002). This signaling step is common for all DSBs independent of the repair pathway choice. The γH2AX-mediated downstream cascade involves such reader and writer enzymes as MDC1, RNF8, and RNF168 (Kolas et al., 2007; Doil et al., 2009). The RNF8-RNF168 ubiquitination cascade recruits main regulators of the repair pathway choice, 53BP1 for NHEJ and BRCA1 for HR. The RNF8-RNF168 axis promotes two chromatin modification marks, which 53BP1 binds in a bivalent mode: the H2AK15ub mark and the H4K20me2 mark (Acs et al., 2011; Mallette et al., 2012; Fradet-Turcotte et al., 2013). Interestingly, the H2AK15 site is a target for both ubiquitination and acetylation and is crucial for repair pathway decisions (Figure 1). When HR is preferred, i.e. in S/G2 phases, the TIP60/NuA4 acetyltransferase complex acetylates H2AK15 and directly blocks ubiquitination which impairs the binding of 53BP1 (Jacquet et al., 2016). The histone acetyltransferase (HAT) TIP60 is also responsible for acetylation of the H4K16 site which physically inhibits 53BP1 binding to H4K20me2 and, thus, promotes HR (Figure 1) (Tang et al., 2013). On the other hand, the removal of H4K16ac from chromatin by the deacetylases HDAC1 and HDAC2 promotes NHEJ (Miller et al., 2010). The removal or absence of methylation at H4K20 (H4K20me0) was reported to guide the pathway choice toward HR by opening the binding site for BRCA1-BARD1, the main HR player (Nakamura et al., 2019). As H4K20me0 is abundant in post-replicative cells, these data provide evidence for how HR can be promoted once the sister chromatid is available as a template. The BRCA1-BARD1 ubiquitinates H2AK127/129 that is read by the ubiquitin reader SMARCAD1 to start a cascade of chromatin remodeling and blocks 53BP1 from binding on damaged chromatin (Densham et al., 2016). There are several chromatin modifications at the DSB sites that mediate the pathway choice in cooperation with the transcription activity of damaged loci, e.g. H3K36me3, H2BK120ac, H2AK118/119ub (Shanbhag et al., 2010; Clouaire et al., 2018). Usually, these modifications are associated with the NHEJ repair pathway but some were shown to be involved in both and additionally regulated by other epigenetic marks or factors (Figure 1).

There are many more chromatin modifications that cooperate at the site of DNA damage to fine-tune the damage signaling, control chromatin remodeling and ensure a successful repair (Figure 1; Table 1). Combined, these data reveal the importance and complexity of chromatin remodeling in DDR.

**TABLE 1:** Histone modifications in the DDR.

PTM	Histone Site	Writer	Eraser	Pathway	References
Phosphorylation	H2AXS139	ATM, ATR, DNA-PKcs	PP2A, PP4, PP6, Wip1	NHEJ, HR	Rogakou et al. (1998)
					Ward and Chen. (2001)
					Stucki et al. (2005)
					Chowdhury et al. (2005), Chowdhury et al. (2008); Douglas et al. (2010); Macůrek et al. (2010)
	H2AXY142	WSTF	EYA1	NHEJ, HR	Xiao et al. (2009)
					Cook et al. (2009)
	H4Y51	TIE2	–	NHEJ	Hossain et al. (2016)
	H4S1	Casein kinase 2	–	NHEJ, HR	Cheung et al. (2005)
					Clouaire et al. (2018)
	H3S10	Aurora-B	–	–	Tjeertes et al. (2009)
	H4T80	Cla4	–	–	Millan-Zambrano et al. (2018)
	H3T45	Akt	–	–	Lee et al. (2015)
	H2BS14	MST1	–	—	Fernandez-Capetillo et al. (2004)
Ubiquitination	H1	RNF8	–	–	Thorslund et al. (2015)
	H2AK13/15	RNF168	OTUB1,USP3, USP11,USP44, USP51,USP26	NHEJ	Fradet-Turcotte et al. (2013); Mailand et al. (2007); Kolas et al. (2007); Mattiroli et al. (2012); Nakada et al. (2010); Sharma et al. (2014); Mosbech et al. (2013); Wang et al.. (2016); Typas et al. (2015); Delgado-Díaz et al. (2014); Yu et al. (2016); Horn et al. (2019)
			USP37,A20, Dub3		
	H2AK118/119	RING1B, FBXL10-RNF68-RNF2	USP16, BAP1	NHEJ	Shanbhag et al. (2010); Rona et al. (2018); Daou et al. (2015)
	H2AXK118/K119	RING1B/BMI1	–	NHEJ	Pan et al. (2011); Wu et al. (2011a)
	H2AK127/129	BRCA1/BARD1	BAP1/ASXL,USP48	HR	Kalb et al. (2014); Densham et al. (2016); Uckelmann et al. (2018)
	H2AZ	RNF168	–	–	O’Connor et al. (2015)
	H2BK120	RNF20/40, UBR7	SAGA	HR	Henry et al. (2003); Nakamura et al. (2011); Moyal et al. (2011); Ramachandran et al. (2016); Dasgupta et al. (2021)
	H4K91	BBAP	–	—	Yan et al. (2009), Yan et al. (2013)
	H2AZK126/K133	–	–	—	Kalocsay et al. (2009)
Acetylation	H2AK15	TIP60	–	HR	Jacquet et al. (2016)
	H2AXK36	CBP/p300	–	–	Jiang et al. (2010)
	H2BK120	SAGA, PCAF	–	HR	Clouaire et al. (2018); Kim et al. (2019)
	H3K9	GCN5, PCAF	SIRT6, HDAC3	–	Tjeertes et al. (2009)
	H3K14	GCN5, PCAF	HDAC3	–	Duan and Smerdon, (2014)
	H3K18	GCN5, p300, CBP	SIRT7	NHEJ	Vazquez et al. (2016)
	H3K56	GCN5,CBP/p300	HDAC1, HDAC2, SIRT1, SIRT2, SIRT6	HR	Masumoto et al. (2005); Tjeertes et al. (2009); Das et al. (2009); Miller et al. (2010); Toiber et al. (2013)
	H4K16	TIP60, MOF, GCN5, p300	HDAC1, HDAC2, SIRT1	HR	Tang et al. (2013); Gong et al. (2015)
Methylation	H2AR3	PRMT7	–	–	Karkhanis et al. (2012)
	H2AXK134	SUV39H2	–	–	Sone et al. (2014)
	H3K4	Set1p	LSD1, KDM5A, KDM5B	NHEJ	Faucher and Wellinger, 2010; Mosammaparast et al., 2013; Li et al., 2014; Gong et al., 2017
	H3K9	KMT1A, DMT1B, SETDB1, PRDM2, SUV39h1	KDM4B, KDM4D	HR	Sun et al., 2009; Young et al., 2013; Khoury-Haddad et al., 2014; Ayrapetov et al., 2014; Khurana et al., 2014; Alagoz et al., 2015
	H3K27	EZH2	–	–	O’Hagan et al. (2008); Campbell et al. (2013)
	H3K36	SETD2, SETMAR	KDM2A, KDM4A	NHEJ, HR	Aymard et al., 2014; Pfister et al., 2014; Carvalho et al., 2014; Fnu et al., 2011; Pai et al., 2014; Amendola et al., 2017
	H3K79	DOT1L	–	NHEJ, HR	Huyen et al. (2004); Wakeman et al. (2012)
	H4R3	PRMT7	–	–	Karkhanis et al. (2012)
	H4K20	KMT5A, KMT5B, KMT5C, SET8	–	NHEJ, HR	Botuyan et al. (2006); Pei et al. (2011); Acs et al. (2011)

## Local and Global Chromatin Changes Mediated by Ubiquitination

Ubiquitination is one of the major chromatin modifications in unperturbed chromatin as well at the sites of DSB in particular. It was discovered as a process of covalent protein modification (Goldknopf et al., 1977) when small chemical modifications like phosphorylation and acetylation were already reported to affect protein properties and function. In the 1980s ubiquitination was shown to be part of the protein degradation pathway *via* the 26S proteasome and for a long time, the proteasomal role of ubiquitin was the only known (Hershko and Ciechanover, 1998). In the last decades, however, an ever-growing number of studies described a non-proteolytical or non-classical role of ubiquitination in intracellular signaling, membrane trafficking, DNA repair, and cell cycle (Dwane et al., 2017; Liao et al., 2022). Through multiple large-scale quantitative proteomic screens, it was confirmed that there are more than 1000 players in the ubiquitin system and more than 10,000 known individual ubiquitination sites in human proteins, which could mean that every cellular protein is ubiquitinated at some point in its existence (Kim et al., 2011; Wagner et al., 2011; Clague et al., 2015). Despite the intense research on ubiquitination, which led to the Nobel Prize in Chemistry for Aaron Ciechanover, Avram Hershko, and Irwin Rose in 2004, there is still a lot unknown about its players and their regulation.

### Ubiquitin-dependent Signaling Network

Ubiquitin (Ub) is a highly conserved 76 amino acids protein, its name due to its massive abundance in all eukaryotic cell types (Goldstein et al., 1975). Ubiquitin is the main building block in ubiquitination, which is a process of covalent attachment of ubiquitin to a target protein. There are four genes encoding ubiquitin in the human genome: UBB, UBC, UBA52, and RPS27A (Kimura and Tanaka, 2010). Translation of these genes does not directly produce free active ubiquitin but requires cleavage of precursors by deubiquitinases (DUB). In addition, DUBs can reverse ubiquitination by cleaving the isopeptide bond between ubiquitin and its substrate protein. In humans there are nearly 90 DUB genes, which can be classified into seven classes (Clague et al., 2019).

**FIGURE 2:**
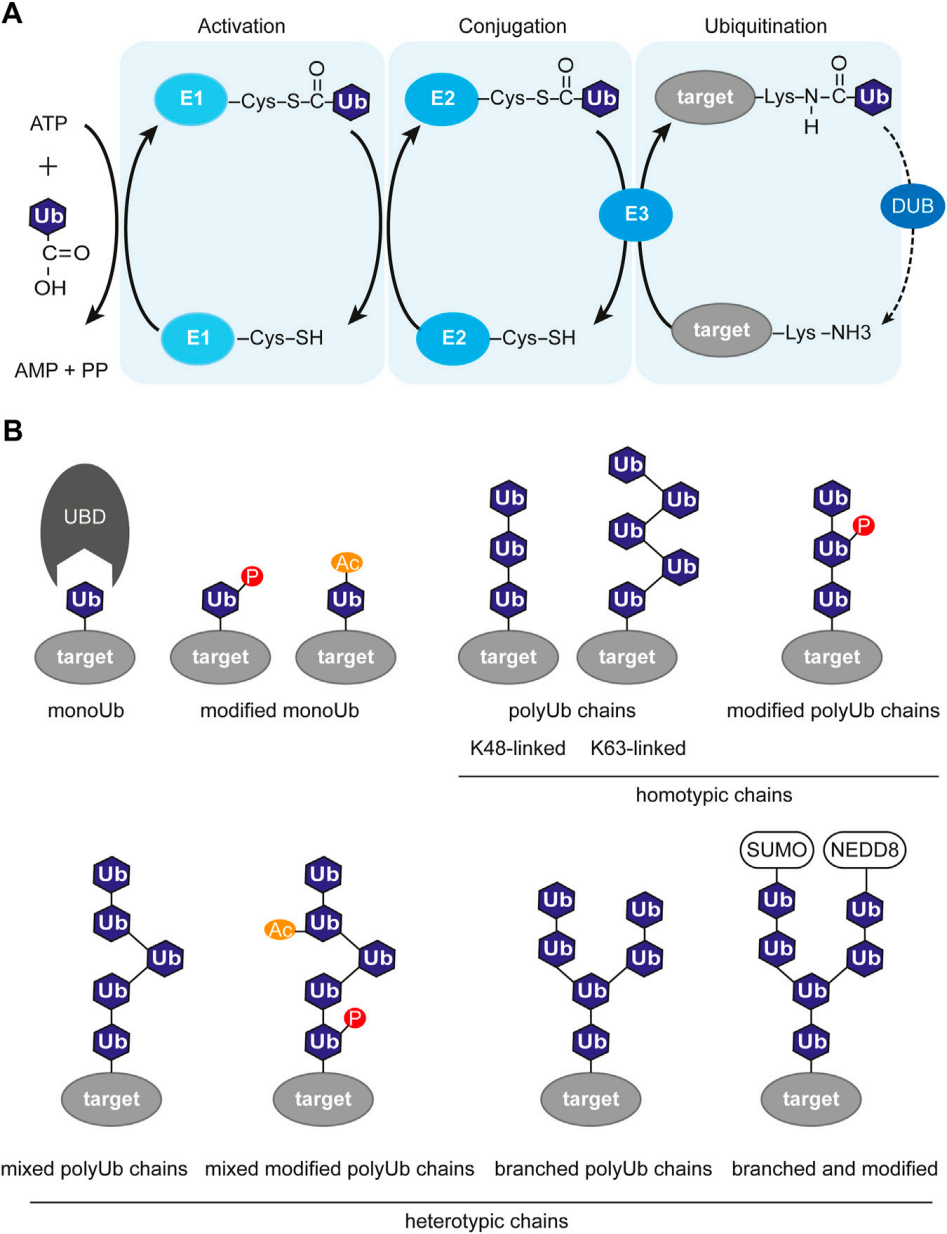
Ubiquitin-dependent signaling system. **(A)** Enzymatic cascade of ubiquitination involving three enzymes. The first step is activation of ubiquitin and covalent attachment to the E1 enzyme. The second step is conjugation, where ubiquitin is transferred to the E2 enzyme. The last step is the transfer of (poly)-ubiquitination to it’s target molecule mediated by a E3 ligase. The ubiquitination process is opposed by deubiquitination, which is the removal of the ubiquitin mark from the target protein by deubiquitinase (DUB). **(B)** Variety of ubiquitin chains, with or without modifications. P, phosphorylation; Ac, acetylation; SUMO, SUMOylation; NEDD8, NEDDylation; Ub, ubiquitin; UBD, ubiquitin-binding domain; target, target protein harboring ubiquitin modification.

The enzymatic cascade of ubiquitination includes three steps and three types of enzymes (Pickart, 2004) (Figure 2A). At first, the free ubiquitin moiety is activated by the E1 enzyme in an ATP-dependent manner by building a thioester bond between the C-terminal Gly carboxyl group of Ub and the Cys in the active site of E1. Then, the activated Ub is delivered to the Cys residue of the E2 conjugating enzyme *via* E1-E2 thioester transfer. The third step is the substrate-specific transfer of the ubiquitin chain to the target molecule. This is achieved by the substrate-specific E3 ligase, which recognizes the E2-Ub complex and catalyzes the formation of an isopeptide bond between the C-terminal carboxyl group of a Ub moiety and an *ε*-NH_2_ group on a lysine on the target protein. There is recent evidence that ubiquitination could also take place on other residues like cysteine, serine, and threonine (McClellan et al., 2019; Mabbitt et al., 2020). There are two ubiquitin E1 genes, around 40 E2, around 90 DUBs, and around 663 E3 ubiquitin ligases genes in the human genome representing around 5% of the total number of genes (Li et al., 2008; George et al., 2018). Interestingly, the number of E3 ubiquitin ligases in the human genome is larger than the number of kinases (518 genes) (Li et al., 2008). The difference in number between the three classes of ubiquitination enzymes evolutionary originates from the ability of one E2 enzyme to cooperate with several different E3 ligases depending on the context, the so-called combinatorial effect. Although there are some E2 proteins able to mediate the direct transfer of Ub to the target, in most cases the E3 ligase is the enzyme that promotes selectivity and specificity of the ubiquitination.

The complexity of the ubiquitin system is also expanded by the fact that ubiquitin itself contains seven lysine residues available for modification: K6, K11, K27, K29, K33, K48, and K63. Multiple ubiquitination events result in the formation of elongated polyubiquitin chains with various structures depending on which lysine was modified (Figure 2B). Apart from the seven internal lysines, there is the eighth site for ubiquitination, which is the methionine at position 1. This results in the formation of non-canonical “linear” chains (Ikeda et al., 2011; Iwai et al., 2014). The topology of the chains defines their function. The K48-linked chains are predominant in cells and usually target their substrate for degradation (Finley, 2009), while K63- and K6-linked chains were shown to regulate DDR (Elia et al., 2015; Takahashi et al., 2018). The wide variety of all the ubiquitination types, like monoubiquitination, multi-monoubiquitination, homotypic polyubiquitination (through the same lysine residue), and heterotypic polyubiquitination (mixed, branched) was named the “ubiquitination code” (Komander and Rape, 2012). The ubiquitin code is steadily expanding with recent findings that ubiquitin itself can be further modified post-translationally by, e.g., phosphorylation at eleven potential sites (Thr7, Thr12, Thr14, Ser20, Ser57, Tyr59, Thr66 (Peng et al., 2003; Lundby et al., 2012; Swaney et al., 2013)), acetylation (Ohtake et al., 2015) and SUMOylation (Hendriks et al., 2014). Those additional Ub modifications may alter its recognition by E3 ligases or Ub-binding proteins. The readers for these modifications are mostly unknown.

The ubiquitination network not only includes writers but also erasers and readers for its function. Some of the proteins might have more than one function and each function is activated by a different signal. While the E3 ligases (RING, HECT, and RBR families) write the ubiquitination and DUBs erase it, the ubiquitination readers are the proteins containing ubiquitin-binding domains (UBD). There are at least 20 types of UBDs present on a wide range of signaling proteins (Dikic et al., 2009). Interestingly, certain types of UBDs are present in proteins enriched at the sites of DSB, namely UIM (ubiquitin-interacting motif), MIU (motif interacting with ubiquitin), and UBZ (ubiquitin-binding zinc-finger) domains (Table 2). The affinity of a single UBD towards ubiquitin marks is low, and, therefore, various mechanisms have evolved to increase the sensitivity and specificity of recognition. Some Ub readers contain multiple UBDs, e.g., the BRCA1-interacting protein RAP80 contains two UIM domains recognizing K63-linked polyubiquitin chains (Sato et al., 2009). Another way for UBD-containing proteins to ensure correct context-specific reading of ubiquitination is to cooperate. Such cooperation was reported for RNF168, RNF169, and RAD18 (Panier et al., 2012), containing bipartite modules composed of UBD and LR motifs (LRM) sequences. Therefore the specific binding is achieved by dual binding of both the ubiquitin binding domain as well as the target specific binding domain. Novel mechanisms of Ub reading and novel UBDs are yet to be identified.

**TABLE 2:** Ubiquitin-binding domains.

UBD Family	UBD	Representative Protein	Ub Epitope	References
α helix	UIM	RAP80	I44	Yan et al. (2007)
	MIU	RNF168	I44	Pinato et al. (2009); Doil et al. (2009)
	UMI	RNF168	I44	Pinato et al. (2011)
	UBA	hHR23a	I44	Wang et al. (2003)
	CUE	FANCD2	I44	Rego et al. (2012)
	GAT	GGA3, TOM1	I44	Prag et al. (2007)
	VHS	STAM, GGA3	—	Wang et al. (2010)
	NUB	NEMO, ABIN1-3	I44	Ea et al. (2006)
Zinc finger	NZF	NPL4, VPS36	I44	Meyer et al. (2002); Sato et al. (2011); Ohkuni et al. (2022)
	ZnF_UBP (PAZ)	USP20, HDAC6	71LRLRGG7, L8, I36	Reyes-Turcu et al. (2006); Yang et al. (2019); Balmik et al. (2021)
	ZnF_A20	ZNF216	D58	Huang et al. (2004)
	UBZ	RAD18, FAAP20	I44	Rizzo et al. (2014); Yang et al. (2010); Toma et al. (2015)
Ubc-like	UEV	BRE, FANCL2, MMS2	I44, Q62	Andersen et al. (2005); Hodson et al. (2011); Rabl et al. (2019)
	UBC	UBE2O, BRUCE	I44	Ge et al. (2015); Huang et al. (2022)
PH domain	GLUE	VPS36	I44	Slagsvold et al. (2005)
	PRU	RPN13	I44 and others	Husnjak et al. (2008)
Others	UBM	Pol ι, REV1	—	Bomar et al. (2010); Niu et al. (2019)
	SH3	BCR/ABL	I44	Slupianek et al. (2009)
	PFU	UFD3	—	Fu et al. (2009)

Taken together, all the variations of the ubiquitin code, alternative Ub modifications, complex networks of regulating proteins, and a large number of putative ubiquitin ligases with still unknown functions constitute a complex, highly dynamic, and largely unexplored ubiquitin-dependent signaling system. Due to its remarkable versatility and exceptional ability to fine-tune a signal transfer, ubiquitination is ideally suited for the regulation of dynamic and complex cellular processes like DSB signaling and repair.

### Local Chromatin Changes: Damage Signaling

The first ubiquitin modifier identified in the DNA damage response was the DNA repair gene RAD6, an E2 enzyme in *S. cerevisiae* (Jentsch et al., 1987). It was also in the DDR where the first evidence of a non-proteolytic function of K63-linked ubiquitination was observed (Spence et al., 1995). Since then evidence has accumulated confirming an essential role of the ubiquitination system in the cellular response to DNA damage.

The first DDR-associated ubiquitination event takes place already within minutes after DNA break induction, and it is the monoubiquitination of histone H2AX at lysine 119/120 by the canonical Polycomb Repressive Complex 1 (PRC1) complex including BMI1-RING1 proteins (Wu C.-Y. et al., 2011; Pan et al., 2011). Additionally, H2A gets ubiquitinated by PRC1 at the same residues and it is believed that this modification induces transcriptional repression, in turn facilitating repair (Chou et al., 2010; Ismail et al., 2010). H2AXK119/120ub modification happens in a DSB-dependent manner and possibly even earlier than the γH2AX formation. It was reported that the H2AXK119/120ub maintains the γH2AX foci stability by directly recruiting ATM to breaks, thus playing a significant role in initiating DNA damage signaling. Additionally, ATM is activated by the MRN complex in the absence of H2AX ubiquitination (Uziel et al., 2003). Since γH2AX is the apical modification event in the reactions cascade of damage response, H2AXK119/120 monoubiquitination subsequently indirectly affects the downstream repair factors. The phosphorylated H2AX is recognized by MDC1 which, when phosphorylated by ATM, tethers both L3MBTL2 and RNF8 to the vicinity of the DNA lesion (Stucki et al., 2005; Nowsheen et al., 2018). At the sites of DSB, the E3 ligase RNF8 performs various types of ubiquitination on different targets: firstly, it polyubiquitinates L3MBTL2 *via* K63-linkage formation, and secondly, histone H2A (Mailand et al., 2007; Stewart et al., 2009; Panier et al., 2012). RNF8-mediated ubiquitination attracts another E3 ligase RNF168 whose activity, in turn, is essential for chromatin rearrangements and recruitment of 53BP1 and BRCA1 *via* K63- and K27-linked ubiquitination (Gatti et al., 2015). Attracted by RNF8-mediated ubiquitination, RNF168 primes monoubiquitination of H2A at lysine 13 and 15 and with the help of RNF8 builds K63-linked chains (Doil et al., 2009; Mattiroli et al., 2012). RNF168 binds modified H2A itself and further accumulates at the DSBs, thereby spreading the ubiquitination. Interestingly, RNF168 activity is mediated not only by recognizing upstream ubiquitination but also by binding an acidic patch on the nucleosome (Leung et al., 2014; Mattiroli et al., 2014). This fact once again confirms how precisely and multitiered the ubiquitination is regulated to ensure the specificity of the signal.

A recent finding revealed another functional layer in the RNF8/RNF168 cascade. It was reported that RNF8 E3 ligase and UBC13 E2 conjugating enzyme mediate the K63-linked ubiquitination of H1 linker histones and, thus, provide a binding platform for RNF168 (Thorslund et al., 2015). This step is required for the further accumulation of ubiquitination at sites of DSBs. The RNF8/RNF168-mediated ubiquitination initiates the recruitment of other ubiquitin modifiers to the vicinity of DNA damage, e.g., HERC2, RAD18, BRCA1, and RNF169 (Huang et al., 2009; Bekker-Jensen et al., 2010; Krais et al., 2021).

Ubiquitination takes the form of a highly regulated dynamic network with multiple interconnected levels of regulation. One layer is the removal of Ub modifications by DUBs. The ubiquitination of H2A at lysine 13/15 is erased by USP51 and USP3 enzymes (Nicassio et al., 2007; Lancini et al., 2014; Sharma et al., 2014; Wang et al., 2016). Other Ub marks catalyzed by the RNF8/RNF168 tandem are removed by USP11 and USP44 DUBs (Mosbech et al., 2013; Yu et al., 2016). Another way to regulate the ubiquitination at DSBs is by controlling the protein levels of E3 ligases. So TRIP12 and UBR5 ubiquitin modifiers negatively control the RNF168 levels by ubiquitin-mediated degradation and, therefore, restrain ubiquitination spreading around damaged chromatin (Gudjonsson et al., 2012), while the RNF8 turnover was reported to be maintained by the VCP/p97/Ataxin complex in a proteasome-dependent manner (Singh et al., 2019). VCP/p97 is an ATP dependent segregase that extracts ubiquitinated proteins from chromatin and shuffles them to the proteasome. Remarkably, the negative regulation of ubiquitination at DSBs can be achieved not only by active removal of the mark or its writers but by changing the message of the signal as well. Accumulation of the K48- and K6-linked chains at the lesion targets proteins to proteasomal degradation, thus controlling the subsequent recruitment of repair factors (Acs et al., 2011; Meerang et al., 2011; Ali et al., 2018). Finally, there is evidence that there might be a non-catalytic competition present at the damage sites. The paralog of RNF168, RNF169 competes for RNF168-generated ubiquitination products with other proteins and, by that, it affects RNF8/RNF168 kinetics and removes 53BP1 from damaged chromatin (Chen et al., 2012; Poulsen et al., 2012; Kitevski-LeBlanc et al., 2017; An et al., 2018).

The RNF8/RNF168 is one of the multiple ubiquitination axes, which take place in DDR. The monoubiquitination of histone H2B at lysine 120 mediated by the RNF20/RNF40 E3 ligases happens in response to DNA damage. The modified H2BK120 attracts the chromatin remodeler SMARCA5 thereby promoting chromatin relaxation and facilitating the recruitment of repair factors (Moyal et al., 2011; Nakamura et al., 2011; So et al., 2019). The removal of the H2BK120ub mark from the chromatin by USP22 and the SAGA complex was reported to regulate the early stage of damage response, however, a lot about this axis is still to be identified.

There is another ubiquitination axis identified that promotes RAP80/BRCA1-A, RAD18, and 53BP1 recruitment to DSBs. Remarkably, it is based on the crosstalk between K63- and K11-polyubiquitin signaling mediated by one reader and eraser protein, Cezanne (Wu et al., 2019). Downstream of RNF8/RNF168, the DUB Cezanne in complex with Cezanne2 binds to K63-linked ubiquitin chains at the sites of damage and removes K11-linked ubiquitination from mixed polyUb chains as well. As the RAP80/BRCA1-A are not able to bind mixed K63-/K11-polyubiquitin chains, the erasure of K11 facilitates its binding.

There are other ubiquitination cascades at the sites of the DSBs but many of them are largely understudied. Not much is known about histones H3 and H4 ubiquitination in the DDR. The CUL4-DDB1-ROC1 complex was shown to ubiquitinate mammalian H3 and H4 histones *in vivo* and *in vitro* in response to UV irradiation (Wang et al., 2006). The homolog of this complex in yeast, however, appeared as a hit in a genetic screen for players of homologous recombination (Moss et al., 2010). Interestingly, the ubiquitination targets of this E3 ligase complex in two different DDR pathways are different as well: while in the nucleotide excision repair (NER) pathway the histones get ubiquitinated, in response to DSBs the CUL4-DDB1-ROC1 complex ubiquitinates the Spd1 protein. This shows that one ubiquitin writer can modify histone and non-histone targets and, therefore, serve various functions in different repair pathways depending on the stimulus.

Another E3 ligase was reported to ubiquitinate histone H4 in response to the DNA damage. BBAP monoubiquitinates H4K91 and protects cells exposed to hydroxyurea and doxorubicin (Yan et al., 2009). Although the levels of γH2AX and MDC1 were not affected in the BBAP knockdown cells, the lack of the H4K91ub mark significantly delayed 53BP1 recruitment to the DSBs. Subsequent investigation of the chromatin marks H4K20me2 and H2AK15 necessary for 53BP1 to bind damaged chromatin showed that their levels were decreased upon the BBAP knockdown. Based on these data, the authors suggested that the H4K91ub is somehow required prior for the H4K20me1/2 and other PTMs to take place at the damage sites, however, the exact mechanism is unknown.

### Local Chromatin Changes: DNA Repair Pathway Choice

Ubiquitination is a fundamental part of DNA damage signaling. DSB repair pathway choice is also modulated by various ubiquitin modifiers. As mentioned above, there are two main DSB repair pathways, NHEJ and HR. The principal difference is reflected in the fidelity of repair, whether or not DSB ends are resected, that is 5′–3′ degradation of one strand at each side of the break. The NHEJ, or mammalian canonical NHEJ (cNHEJ), is a very fast repair pathway that employs end ligation with minimum resection and sequence homology. In contrast, the multi-step HR is driven by homology search and broken ends resection, where single-strand DNA uses an intact copy of the damaged locus as a template for repair and, therefore, requires significant homology between sequences. As a result, this difference leads to the concept of HR being the pathway that provides the most accurate repair of DSB, while NHEJ might serve as a source of point mutations and small deletions.

The correct choice of repair pathway at every DSB is of utmost importance for cell survival. Both pathways co-exist in a cell at the same time and partially compete for the DSBs (Beucher et al., 2009). Multiple factors affect the repair pathway decision in a spatio-temporal manner. One of the approaches suggested structuring our knowledge about all these factors and their coordination with each other in a way we could predict the pathway choice in a so-called “decision tree” (Scully et al., 2019). The first factor which affects the pathway choice is open DNA ends themselves. Depending on the source of damage and initial chromatin state, the composition of the break may significantly vary, which in turn affects the binding affinity of damage sensor proteins, e.g. Ku70/80 shows weak binding affinity to long ssDNA (Mimori and Hardin, 1986). The Ku70/80 binding to the break initiates NHEJ, while the displacement of the Ku70/80 heterodimer from the broken DNA ends is a necessary step to initiate resection and turn the repair in direction of HR (Langerak et al., 2011). The Ku70/80 removal from the ends can be achieved by ubiquitin-mediated degradation *via* K48-linked chains added to Ku70 (Postow et al., 2008; Ismail et al., 2015), followed by VCP/p97 mediated extraction from chromatin. Another important factor in the pathway choice is the cell cycle stage (Hustedt and Durocher, 2016). The NHEJ is functional throughout the cell cycle while the HR is a preferable choice in the S-G2 stages when a sister chromatid is available for template-guided repair, therefore the cell cycle stage is directly related to the resection (Mao et al., 2008). The cell cycle status can be directly transmitted from the cell cycle-dependent kinases (CDK) to the repair machinery, e.g. through phosphorylation of resection players CtIP and EXO1 (Tomimatsu et al., 2014; Anand et al., 2016). In G1 cells instead, CtIP gets ubiquitinated and targeted for degradation by the anaphase-promoting complex APC/C thus blocking resection (Lafranchi et al., 2014). Recently another way for the APC/C complex to regulate pathway choice in a ubiquitin-dependent manner was identified. The authors showed that in G1 cells BRCA1 recruitment to DSB is obstructed due to the USP1-mediated removal of K63-linked polyubiquitin chains from histones. In contrast, in S-G2 phases, USP1 is ubiquitinated by the APC/C complex and degraded. This process requires Chk1 activation as well (Ha et al., 2017). Aside from USP1, there were more DUB enzymes identified lately that modulate pathway choice by facilitating retention of resection proteins CtIP and EXO1 at the sites of damage, namely USP4 and UCHL5 (Liu et al., 2015; Wijnhoven et al., 2015).

**FIGURE 3:**
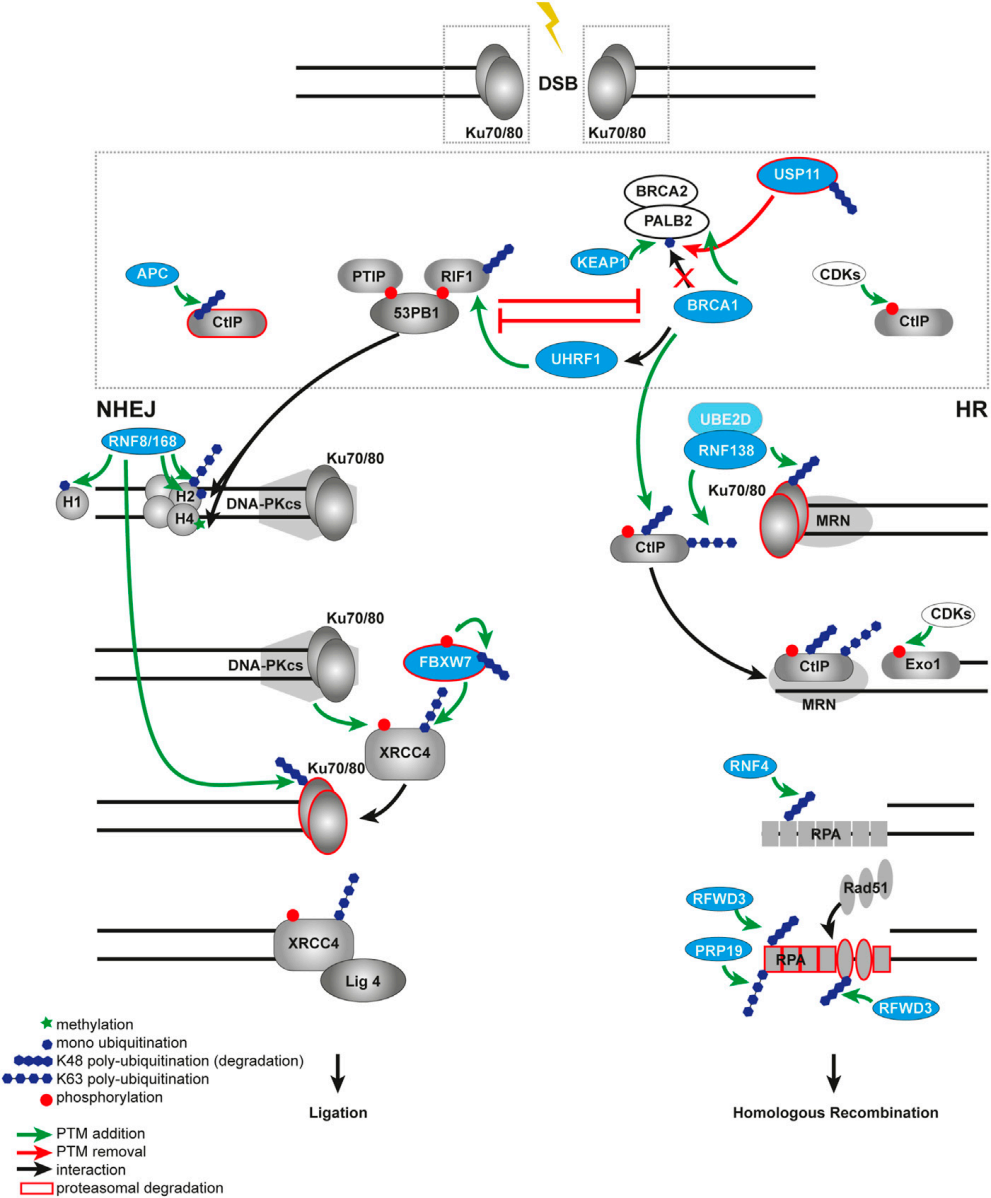
Ubiquitination in DSB repair pathway choice. Open DNA ends are rapidly recognized by the Ku70/80 heterodimer. The choice between NHEJ and HR at the DSB is controlled by the competition between 53BP1 and BRCA1. In a NHEJ-permissive environment, 53BP1 builds a complex *via* ATM in a phosphorylation-dependent manner with its binding partners RIF1 and PTIP. The 53BP1-PTIP-RIF1 complex prevents recruitment of BRCA1 to the break and, therefore, antagonizes HR. The E3 ubiquitin ligase KEAP1 ubiquitinates PALB2 at the BRCA1 binding site and prevents assembling of the BRCA1-BRCA2-PALB2 complex, thus, promoting NHEJ. This process is balanced by the DUB USP11 and controlled by the cell cycle. BRCA1 antagonizes NHEJ by impairing retention of RIF1 at DSB sites in a CtIP- or UHRF1-dependent manner. Ubiquitin-dependent degradation of CtIP by the APC/C in NHEJ prevents resection process, in contrast to HR, where CtIP is not degraded but phosphorylated by the CDKs. The RNF8-RNF168-mediated ubiquitination on chromatin provides a binding platform for both 53BP1 and BRCA1. Later RNF8 also ubiquitinates Ku70/80 to facilitate its proteasomal degradation and removal from the broken DNA ends. In a HR-permissive environment the E3 ubiquitin ligase RNF138 ubiquitinates Ku to remove it from DSB much earlier compared to NHEJ, allowing end-resection factors to gain access to the deprotected ends. RNF138 also ubiquitinates CtIP, enhancing its retention on damaged chromatin. At the later steps of NHEJ the ligation of broken ends promoted by FBXW7 that recognizes phosphorylated XRRC4 and subsequently ubiquitinates it. In contrast during the later steps of HR, RPA SUMOylation-guided ubiquitination by RNF4 promotes RPA turnover and RAD51 recruitment. Multi-site ubiquitination of RPA by the ubiquitin ligases RFWD3 and PRP19 also facilitates HR.

Another ubiquitin modifier that affects repair pathway choice is the E3 ligase RNF138 (Figure 3). Interestingly, RNF138 promotes HR by ubiquitination of two substrates. First, RNF138 ubiquitinates CtIP and causes its retention at DSBs, second, RNF138 ubiquitinates the Ku80 subunit of the Ku70/80 heterodimer which results in Ku70/80 displacement (Ismail et al., 2015; Schmidt et al., 2015). The ubiquitination of Ku80 happens in the S phase only and, thus, there must be an additional cell cycle-dependent regulation mechanism.

The RNF138-mediated dual ubiquitination of two different targets in one process is an important example of the versatility of the ubiquitin-dependent network in DDR. Another example is when one target can be ubiquitinated by multiple different writers, e.g. the Ku70/80 can be removed from DSB ends by various ubiquitin E3 ligases. The RNF8 and SCF-FBXL12 ligases promote ubiquitination and subsequent removal of Ku70/80 as well (Feng and Chen, 2012; Postow and Funabiki, 2013).

At the sites of DSBs, 53BP1 and BRCA1 proteins antagonize each other in repair pathway choice. It was shown that both of them are recruited to the break *via* the RNF8/RNF168 ubiquitination cascade. We discussed earlier that 53BP1 uses bivalent recognition of both RNF8/RNF168-mediated H2AK15ub ubiquitination and H4K20me2 methylation to bind to damaged chromatin. In undamaged cells, the methylation mark is hidden from 53BP1 interaction by KDM4A (JMJD2A) and L3MBTL1, but at DSB sites both of them are targeted to proteasomal degradation (Acs et al., 2011; Mallette et al., 2012; Fradet-Turcotte et al., 2013). Once bound to chromatin in G1, 53BP1 promotes NHEJ through the recruitment of its interaction factors telomere-associated protein RIF1, PTIP, Shieldin and CST complex and REV7 and suppression of BRCA1 (Chapman et al., 2013; Di Virgilio et al., 2013; Wang et al., 2014; Xu et al., 2015; Mirman et al., 2018; Noordermeer et al., 2018). Moreover, a new ubiquitin-dependent mechanism for suppression of HR in G1 cells was identified (Orthwein et al., 2015). The ubiquitination of PALB2, one of the interacting partners for BRCA1, by KEAP1 E3 ligase complex blocks its interaction with BRCA1 and thus HR in G1 cells (Figure 3) (Orthwein et al., 2015). The DUB USP11 that removes ubiquitin from PALB2, promoting BRCA1–PALB2–BRCA2 complex formation in S/G2 cells, opposes this process (Schoenfeld et al., 2004; Orthwein et al., 2015). Interestingly, recent study described the role of USP11 in HR in centromeric regions in G1 in the absence of the sister chromatid and taking advantage of centromeric transcripts forming DNA-RNA hybrids. The DUB USP11 was shown to promote the recruitment of the RAD51-BRCA1-BRCA2 complex to centromeres by interaction with the centromeric histone CENP-A and HJURP (Yilmaz et al., 2021). In contrast, the HR-favoring conditions are characterized by ubiquitination of histone H2A at K125/127/129 by BRCA1-BARD1 (Kalb et al., 2014). The H2AK125/127/129ub in response to DSBs supports SMARCAD1 recruitment, which in turn facilitates repositioning of 53BP1 to the periphery of the repair focus, and promotes resection (Densham et al., 2016). Later the same group also identified the DUB USP48 for negative regulation of BRCA1-mediated resection (Uckelmann et al., 2018). The very recent studies further expanded our knowledge about the BRCA1-BARD1 repair mechanism in post-replicative chromatin (Becker et al., 2021; Hu et al., 2021). With help of cryo-electron microscopy, the authors revealed that BRCA1-BARD1 specifically binds to nucleosomal histones, DNA, and H2AK13/K15 monoubiquitination at the DSB sites and subsequently promotes ubiquitination at the C- terminus of H2A (Hu et al., 2021). *Via* multivalent interactions BARD1 mediates recruitment of BRCA1 to ubiquitinated chromatin, but other ubiquitin modifiers control the BARD1 protein itself. Newly identified E3 ligase RNF19A ubiquitinates BARD1 to prevent its retention and homologous recombination in the NHEJ-favored chromatin context (Zhu et al., 2021). Even more mechanisms of how BRCA1 antagonizes 53BP1 were identified. Several studies reported that BRCA1 impairs the retention of 53BP1 interacting partner RIF1 at the damaged chromatin in the S phase in either a CtIP-dependent manner (Escribano-Díaz et al., 2013) or *via* UHRF1 E3 ligase recruitment (Zhang H. et al., 2016). Interestingly, CtIP ubiquitination by BRCA1 does not result in its degradation but instead strengthens its role in the G2/M checkpoint. BRCA1 recruitment to DSBs was shown to be mediated by deubiquitination events as well (Li et al., 2017). The DUB enzyme USP13 deubiquitinates RAP80 which facilitates its interaction with BRCA1 and subsequent recognition of the K63-ubiquitinated chromatin (histones) in the vicinity of DSBs.

Ubiquitination affects DNA repair pathway choice at all steps of DDR. For example, during RPA-coated ssDNA formation and homology search, the E3 ligases PRP19 and RFWD3 control the stability of RPA and RAD51 nucleofilaments, thus, promoting HR (Figure 3). At the sites of DNA damage, RFWD3 polyubiquitinates both RPA and RAD51 in a phosphorylation-dependent manner, which requires the help of ATM and ATR (Dubois et al., 2017; Inano et al., 2017). In this case, VCP/p97 mediates eviction of RPA and RAD51 polyubiquitinated by RFWD3 from early DSB repair sites for proteosomal degradation. The E3 ligase PRP19 was reported to polyubiquitinate RPA *via* K63 linkages and promote ATRIP accumulation and subsequent ATR activation (Maréchal et al., 2014). Together these ubiquitin modifiers support late-HR progression in time and space.

Despite the seeming abundance of our knowledge about ubiquitination-guided repair pathway choice, a lot of questions remain unanswered: are there novel and yet undiscovered ubiquitin modifiers in DDR and what steps do they regulate; how do ubiquitin modifiers interact with DSB signaling network; how does ubiquitin code modulate DDR signaling etc. There is no doubt that a lot of new ubiquitin modifiers and regulation mechanisms are to be identified in the future.

The ubiquitination events are omnipresent throughout all steps of DDR from damage recognition to successful repair of the lesion. Ubiquitination signaling orchestrates DDR not only locally at the site of broken ends but also participates in the formation of a repair domain at a larger scale.

### Global Chromatin Changes

Chromatin domains affect DSB formation and get affected by it at multiple hierarchically organized structural levels. On higher folding levels newly built breaks initiate the formation of repair domains, microscopically visible as foci, in the case of radiation known as radiation-induced foci (IRIF). The foci can be visualized by immunofluorescent labeling of various damage signaling and repair proteins but the most important for chromatin structure study is the labeling of γH2AX. The phosphorylation of H2AX spreads far beyond the actual break location over megabase-pair distance (Rogakou et al., 1999). It is generally believed that every single focus at the conventional microscopy level represents one break and the large spreading of phosphorylation was an intriguing fact back then. There is a lot of evidence that chromatin architecture controls the formation of the γH2AX-decorated repair domain. The super-resolution light microscopy showed that CTCF protein, which restrains cohesin-mediated loop extrusion and therefore essentially shapes the 3D organization of large chromatin domains, is juxtaposed to γH2AX foci. Moreover, the depletion of CTCF heavily impaired DDR signaling and repair efficiency in cells subjected to radiation (Natale et al., 2017). Other studies utilizing endonuclease AsiSI for targeted DSB generation in mammalian cells showed NIPBL-, MRN-, and ATM-mediated accumulation of cohesin at the DSB site (Arnould et al., 2021). With help of chromosome conformation capture mapping and chromatin immunoprecipitation (ChIP) assays, the authors revealed a surprising discrepancy between γH2AX spreading and accumulation of its kinase ATM around the break that suggests that H2AX phosphorylation is not just a linear, ATM-dependent process. Their study highlights the one-sided loop extrusion on either side of the break as a prime mechanism of γH2AX domain formation. Also, chromatin structural domains (TAD, topologically associated domain) are a template for the repair domain (Arnould et al., 2021). Thus, DDR utilizes a large-scale chromatin structure remodeling for genome maintenance.

Interestingly, similar to γH2AX a cohesin-dependent arrangement on chromatin was reported for other repair proteins like 53BP1, ubiquitination events, and H1 histone eviction (Clouaire et al., 2018; Ochs et al., 2019). Remarkably, ubiquitination of damaged chromatin follows γH2AX spreading almost precisely (Clouaire et al., 2018), not being restricted to the vicinity of the break. It is evident now that various histone/non-histone ubiquitin modifications and ubiquitin modifiers contribute as well to the formation of the 3D repair-permissive domain to facilitate the repair and, in the end, to maintain chromatin topology at sites of DNA breakage.

The chromatin domains vary in their properties. Pre-existing heterochromatin allows the entrance of some macromolecules and chromatin remodelers, e.g. due to the formation of a phase-separated domain with selective permeability (Gibson et al., 2019; Wang et al., 2019). Liquid-liquid phase separation (LLPS) as a force driving and maintaining chromatin compartmentalization is promoted by the chromatin scaffold, histone modifications, and their multiple reader proteins (Snead and Gladfelter, 2019). A newly formed lesion starts a cascade of changes in the chromatin context, which leads to its reorganization at a large scale to form a 3D domain outlining a repair-prone environment. In recent years, a growing number of publications highlighted the role of phase separation in DNA damage response. All levels, initiation, spatial organization/clustering of damage, maintenance of damage domain 3D architecture, and repair are affected (Kilic et al., 2019; Pessina et al., 2019; Singatulina et al., 2019; Oshidari et al., 2020; Frattini et al., 2021; Ghodke et al., 2021; Levone et al., 2021; Miné-Hattab et al., 2021). Remarkably, PTMs, namely ubiquitination and its crosstalk play a significant role in it. For example, it was proposed that 53BP1-seeded phase separation requires both interaction between 53BP1 and H4K20me2, as well as 53BP1 interaction with RNF168-mediated, DNA damage-induced H2AK15 ubiquitin marks (Kilic et al., 2019). When the 53BP1 LLPS is affected, the downstream DNA repair is impaired. Therefore modifiers like RNF169, TRIP12, and UBR5 that antagonize the RNF168-mediated ubiquitination spreading, indirectly control phase separation at the sites of the DSBs. Another seeding factor for the phase separation in DDR is poly(ADP-ribosyl)ation (PARylation), which is mediated by PARP1 activation. It was shown before that PAR chains can synergize with various intrinsically disordered proteins, e.g. FUS, to initiate LLPS, however, a recent study described a DUB enzyme to bind PAR chains in a damage-dependent manner (Kim et al., 2021). The USP39 deubiquitinase is recruited to the DSBs by the poly(ADP-ribosyl)ation with help of its tripartite RG domain, thus inducing liquid demixing and promoting NHEJ. The authors also suggested that the USP39 function is required for HR, however, it is not related to PAR or phase separation. Additionally, the USP39 activity is an important example of PAR-ubiquitination crosstalk in the DDR.

The ubiquitination and other PTMs on histones and non-histone proteins not only change the chemical properties of the target proteins but could also control the abundance of other molecules like RNA in the phase-separated domain. However, our understanding of how ubiquitination contributes to the phase separation in damaged chromatin is only starting to form, and more studies are needed to uncover this network.

It is exciting to think about how phase separation integrates with active molecular processes at the DNA damage sites. Although some repair proteins like 53BP1 are observed to initiate the phase separation, upstream γH2AX and MDC1 accumulation do not seem to be involved in it (Kilic et al., 2019). This fact hints that the formation of the repair domain is a product of multiple different processes, and their interplay is yet to be investigated.

Chromatin responds dynamically to the damage. Upon DNA damage, mammalian and yeast chromatin domains go through decompaction and expansion, which in turn changes the mobility and accessibility of the damaged locus. It is well documented now that the mobility of damaged locus increases, reflecting exploration of the nuclear space by DSB end during “homology search” (Hauer and Gasser, 2017; Herbert et al., 2017; Miné-Hattab et al., 2017). However, undamaged loci in damaged cells were observed to elevate their mobility as well but to a lesser extent, which points out that changes in chromatin mobility are a general feature of the whole-genome response to DSBs (Chiolo et al., 2011; Miné-Hattab et al., 2017; Caridi et al., 2018; Smith et al., 2019). Moreover, the DSBs in *Drosophila* and mouse cells were observed to relocalize to subnuclear domains facilitating repair: from pericentromeric heterochromatin regions to the nuclear periphery in flies and from the core to the outer layer of chromocenters in mice (Chiolo et al., 2011; Jakob et al., 2011; Tsouroula et al., 2016). Tsouroula et al. and Natale et al. additionally showed that damage-induced global expansion in centromeric and pericentromeric domains was not accompanied by the removal of silent chromatin marks and was not connected to the relocalization happening in mammals (Tsouroula et al., 2016; Natale et al., 2017). This finding supported the previously stated integral function of localized recompaction in checkpoint signaling but not in repair (Burgess et al., 2014). This suggests that chromatin compaction may regulate the timing of different steps of the DDR. In contrast to pericentromeric DSBs, centromeric DSBs were not shown to relocalize. Instead, they trigger new deposition of centromeric histone variant CENP-A, which allows the specific recruitment of DUB USP11 to damaged centromeres to facilitate RAD51 engagement and initiation of HR (Yilmaz et al., 2021).

The transient chromatin decompaction occurs at the sites of DSBs independently of the repair pathway. Such decompaction and accompanying it increased mobility correlated to significant depletion of nucleosomes on DNA with proteolytic degradation of 30–40% of core histones in yeast (Hauer et al., 2017). A similar effect was shown when core histone reduction was achieved by deletion of genes coding for histones H3 and H4 (Liang et al., 2012). Recent work of the Gasser group further explored the chromatin remodeling effect mediated by histone depletion and revealed the paramount role of ubiquitin ligases and chromatin ubiquitination in the mobility of the damaged site in yeast (Cheblal et al., 2020; Challa et al., 2021). They showed that five ubiquitin-conjugating factors are recruited to the damage sites in a checkpoint- and INO80C-dependent manner which results in depletion of core and H1 histones up to 20–40%, chromatin decompaction, and enhanced DNA locus mobility (Challa et al., 2021). Remarkably, a homolog of one of them was proposed to cause chromatin decompaction in mammalian cells as well. The RNF20/40 ligases (Bre1 in yeast) promote the formation of the H2BK120ub mark in damaged cells, which facilitates chromatin relaxation and repair proteins recruitment by promoting nucleosome disassembly (Chernikova et al., 2010; Moyal et al., 2011; Nakamura et al., 2011; Zheng et al., 2018; So et al., 2019; Liu et al., 2021). Interestingly, the monoubiquitination of histone H2B itself directly impairs the compaction of higher-order chromatin structures and results in open conformation (Fierz et al., 2011). The mechanism is different from the acetylation-mediated decompaction and in contrast to it, monoubiquitination spreads over larger distances. Lastly, the H2BK120ub was shown to crosstalk with other chromatin modifications at the damage sites, namely H4K20 and H4K9 methylation, which could lead to a pleiotropic chromatin reorganization.

Speaking of chromatin mobility in DDR, we cannot ignore the clustering of DNA damage. The clustering is thought to be happening to DSBs which require resection of broken ends or concentration of several damaged sites into the large domain to facilitate repair (Aymard et al., 2017; Schrank et al., 2018; Schrank and Gautier, 2019). It was recently identified that actin filament nucleators are the driving force for the DSB clustering and are a factor in the repair (Schrank et al., 2018). At last, the clustering can also protect DSBs from improper repair, where one of the examples is coordination between end resection degree and amount of damage inflicted on cells.

## Crosstalk Between Ubiquitination and Other Chromatin Modifications in DDR

Crosstalk between various PTMs on histone and non-histone proteins lies at the basis of DNA damage signaling and repair. A single PTM affects the properties and function of its target, but only coordination of phosphorylation, acetylation, methylation, poly(ADP-ribosyl)ation, ubiquitination, and their readers ensure the spatiotemporal recruitment of repair proteins and chromatin remodeling at the sites of damage. There are several classifications of crosstalk proposed, e.g. based on how modifications are hierarchically organized in a cascade (Dantuma and van Attikum, 2016). According to it, the PTMs can be serial, when one modification sequentially promotes other modifications; parallel, when one reader containing multiple binding domains recognizes multiple independent modifications; and combinatorial, when two or more modifications are equally required for one reader to induce a signal. Above, we described some of the examples of the ubiquitin-dependent regulatory cascades, but the ubiquitination crosstalk with other PTMs constitutes an integrated signaling network in DDR.

### Phosphorylation and Ubiquitination

**FIGURE 4:**
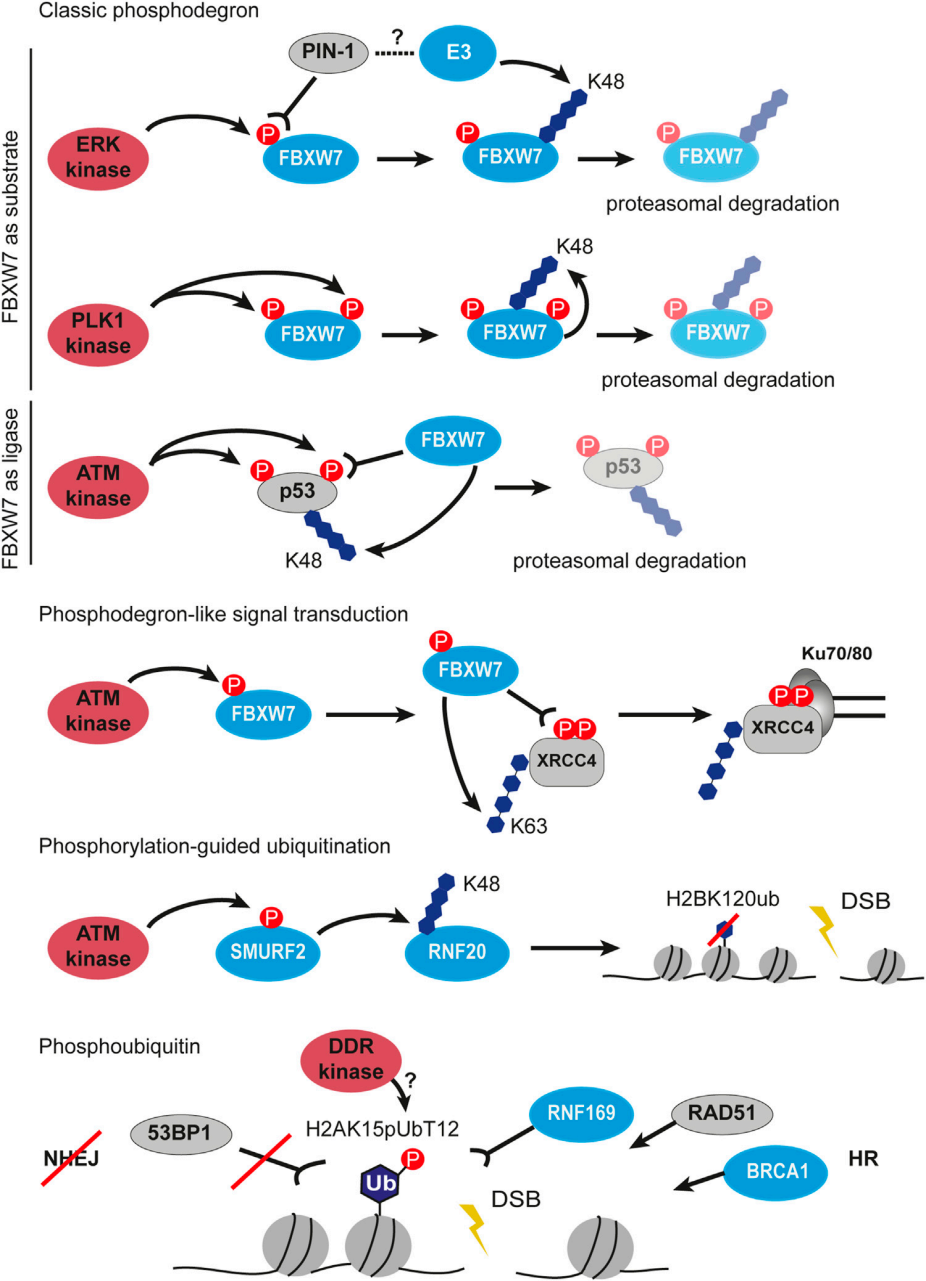
Crosstalk between phosphorylation and ubiquitination in DSB signaling and repair. First three panels describe the classic phosphodegron situations highlighting FBXW7 as substrate to phosphorylation and as phosphorylation reading ubiquitin ligase. Next, phosphodegron-like signal transduction by FBXW7 is shown, which results in ubiquitination and stabilization of XRCC4 at Ku70/80 covered DSB. The next panel depicts phosphorylation-guided ubiquitination involving sequential signal transfer by two ubiquitin ligases SMURF2 and RNF20. The last example of the crosstalk between phosphorylation and ubiquitination is the phosphorylation of the ubiquitin moiety. The H2AK15pUbT12 is recognized by the HR factors such as RNF169, RAD51 and BRCA1, but prevents binding of 53BP1 at DSB. P, phosphorylation, Ub, ubiquitin.

Phosphorylation is among the most abundant and most studied modifications at the sites of DNA damage. Crosstalk between phosphorylation and ubiquitination is essential for the correct execution of DDR. One of the examples of this crosstalk is phosphorylation-guided proteasomal degradation with help of phosphodegrons. Phosphodegrons are one or several residues on a target protein that can be phosphorylated by a kinase and subsequently bound by an E3 ubiquitin ligase to initiate proteasomal degradation *via* K48-linked ubiquitin chains (Ang and Wade Harper, 2005). At the sites of DSB, the NHEJ E3 ligase FBXW7 stability is controlled by phosphorylation and the creation of the phosphodegron signal (Figure 4). The extracellular signal-regulated kinase (ERK) directly interacts with and phosphorylates FBXW7 at Thr205, which results in the degradation of FBXW7 in a PIN-1-dependent manner by an unknown E3 ligase (Ji et al., 2015). When FBXW7 is phosphorylated at Ser58 and Thr284 by kinase PLK1, it autoubiquitinates itself which also leads to its degradation (Xiao et al., 2016). Remarkably, FBXW7 can not only be a substrate for simultaneous phosphorylation and ubiquitination but also acts as the degradation-inducing E3 ligase for other substrates, e.g. p53 (Figure 4) (Cui et al., 2020). In response to etoposide-induced DNA damage, ATM phosphorylates p53 on Ser33 and Ser37, which facilitates the FBXW7 binding and subsequent p53 degradation. The knockdown/knockout or chemical inhibition of FBXW7 increased p53 protein half-life upon DNA damage in an MDM2-independent manner, therefore sensitizing cells to radiation and etoposide treatment. Thus, the phosphorylation and ubiquitination crosstalk is finely tuned to ensure the protection of genome stability. Importantly, the “phosphodegron” signal does not necessarily result in degradation (Figure 4). Phosphorylated by ATM at Ser26, FBXW7 is recruited to the sites of DSB, where it recognizes another phosphorylated protein XRCC4. Upon phosphorylation at Ser325/326 by DNA-PKcs, XRCC4 is bound and ubiquitinated by FBXW7 *via* K63-chains at lysine 296 (Figure 4). The K63-linked polyubiquitination of XRCC4 enhances its association with the Ku70/80 complex to facilitate NHEJ repair (Zhang Q. et al., 2016). Recently, a systematic analysis of MARKs-phosphorylated degron motifs recognized by FBXW7 identified new targets among chromatin proteins (Singh et al., 2022). The exact mechanism remains elusive, however, it provides a look at the large regulation network managed by phosphorylation and ubiquitination.

Phosphodegron cascades are possible due to the proteins containing both phosphorylation and ubiquitination sites. A comprehensive statistical analysis of the sequence and spatial distributions of PTMs revealed that among all the PTM pairs studied, phosphorylation and ubiquitination sites have a smaller distance between each other on the same proteins compared to random distance distribution (Korkuć and Walther, 2017). This close proximity of PTM sites is encoded in the protein sequence and evolutionary conserved. The close vicinity allows regulatory interference between ubiquitination and phosphorylation by steric interaction/hindrance. For example, ubiquitination sites enrichment near the domain activation loop and in the glycine-rich region of kinases were reported to serve for reversible inhibition (Swaney et al., 2013). The same proteome-wide study identified 466 yeast proteins with 2100 phosphorylation sites co-occurring with 2189 ubiquitination sites using mass spectrometry-based methods. Interestingly, greater conservation of phosphorylation on ubiquitinated proteins was observed than the other way around, which highlights the existence of crosstalk directionality where phosphorylation tends to precede ubiquitination (Swaney et al., 2013).

One of the central phosphorylation events in DDR is γH2AX formation mediated by ATM, ATR, and DNA-PKcs. Above we have already discussed in detail the γH2AX-dependent chromatin-associated RNF8/RNF168 ubiquitination axis (Kolas et al., 2007; Doil et al., 2009). Yet ATM phosphorylates various substrates in various damage response contexts and subsequently promotes various multifaceted ubiquitination events. Together with the CHK2 kinase, ATM phosphorylates CABIN1 which acts as a negative regulator of p53 activity. The phosphorylated CABIN1 is recognized and targeted to the ubiquitin-dependent proteasomal degradation mediated by the CRL4-DDB2 ubiquitin ligase complex, thus activating p53 in response to the genotoxic stress (Choi et al., 2013). There are more negative regulators of p53 at the damage sites, HDMX (human ortholog of Mdmx) is one of them as well. It was shown that in response to the lesion ATM phosphorylates HDMX at Ser395 and promotes its degradation by the E3 ligase HDM2 (human ortholog of Mdm2) (Pereg et al., 2005). The precise regulation of the ATM-mediated p53 activation and stabilization remains elusive due to the abundance of the phosphorylation-ubiquitination circuits. It was recently reported that the E3 ligase Mdm2 can be phosphorylated by ATM as well which leads to its autoubiquitination and subsequent degradation (Magnussen et al., 2020). More evidence is required to fill the gap in our knowledge about the organization of separate cascades and their crosstalk within the DDR network.

Apart from the p53 regulation, ATM was observed to phosphorylate Ser384 on the E3 ligase SMURF2, an interaction partner of RNF20 (Figure 4) (Tang et al., 2020). In turn, phosphoSMURF2 binds and ubiquitinates RNF20 promoting its proteasomal degradation. The SMURF2-mediated RNF20 ubiquitination results in a decrease of H2BK120 ubiquitination, thereby promoting chromatin compaction and protecting cells from DNA damage. However, in the absence of phosphorylation or SMURF2 knockout, the repair was shown to proceed faster than in wild-type cells as measured by γH2AX disappearance, probably, due to excessive relaxation of chromatin and facilitated recruitment of repair proteins. Despite faster DSB repair, the mouse embryonic fibroblasts with the mutant SMURF2 showed higher sensitivity to etoposide treatment which means that timely chromatin remodeling is of utmost importance for DDR (Tang et al., 2020).

Phosphoubiquitin (pUb) was shown for the first time to play a role in DNA damage signaling and repair only recently (Figure 4). Walser and colleagues described that the ubiquitin moiety phosphorylated at Thr12 (pUbT12) is used for the formation of a new chromatin mark H2AK15pUbT12 (Walser et al., 2020). Authors reported that the H2AK15pUbT12 levels increase in response to the radiation- and etoposide-induced DSBs and return back to the basal state after DNA repair. The H2AK15pUbT12 is differently recognized by ubiquitin readers in DDR: the phosphorylation of H2AK15ub abolishes 53BP1 binding while being still bound by RNF169. Thus, chromatin modified by pUbT12 is inaccessible to 53BP1 but permissive to HR proteins like RNF169, RAD51, and the BRCA1/BARD1 complex, which makes the H2AK15pUbT12 an HR-specific chromatin modification. Interestingly, the pUbT12 prevents the removal of the H2AK15pUbT12 mark by DUB USP51 from chromatin which results in the retention of ubiquitination and formation of the HR-prone environment. The DDR kinase responsible for this phosphorylation on ubiquitin is unknown as well as its mechanism, although the authors revealed that it depends on preceding ubiquitination events mediated by RNF168 (Walser et al., 2020). Taken together, the phosphoubiquitin modifications represent a new type of DDR signaling.

### Poly(ADP-Ribosyl)ation and Ubiquitination

Poly(ADP-ribose) (PAR) is a PTM that consists of at least two or more ADP-ribose molecules covalently linked by glycosidic ribose–ribose bonds. Formation of the poly(ADP-ribose) chains or PARylation can occur at different amino acid residues, including aspartate, glutamate, and lysine residues, mediated by the family of the poly(ADP-ribose) polymerases (PARPs) (Leung, 2014). At the sites of DNA damage, the most abundant PARP is poly(ADP-ribose) polymerase 1 (PARP1) whose recruitment results in rapid and reversible PARylation at histone, non-histone proteins, and PARP1 itself (Tallis et al., 2014).

**FIGURE 5:**
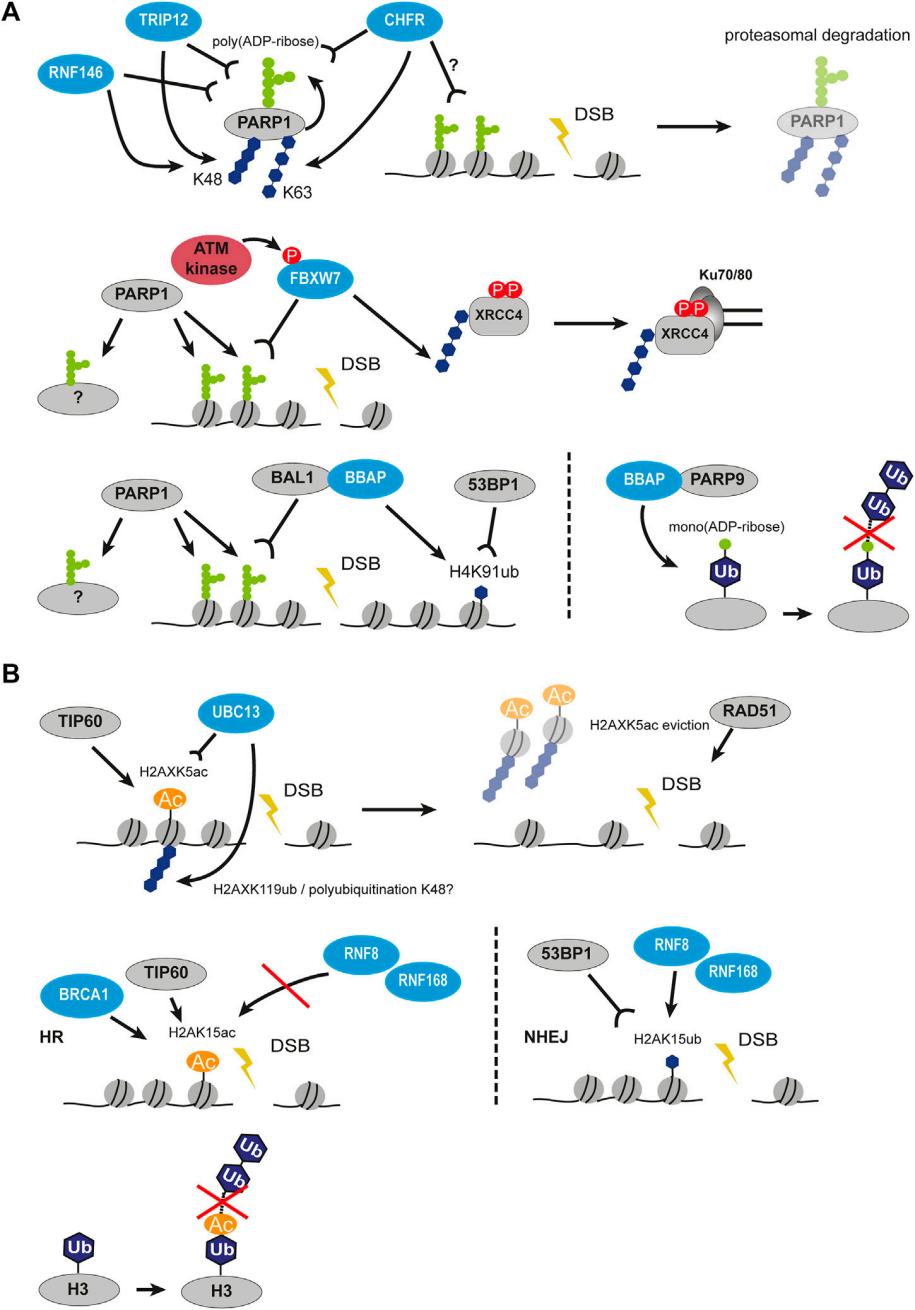
Crosstalk between PARylation and ubiquitination, as well as acetylation and ubiquitination in DSB signaling and repair. **(A)** Crosstalk between PARylation and ubiquitination. PARP1 auto-PARylates itself followed by recognition of PAR chains by several ubiquitin ligases such as CHFR, RNF146, TRIP12 and subsequent polyubiquitination. This results in proteasomal degradation of PARP1. PARP1-mediated modification of histones at DSBs can be recognized by FBXW7, which polyubiquitinates XRRC4 and leads to its interaction with Ku70/80. On the other hand, PARP1-mediated modification of histones can also be recognized by BAL1-BBAP which leads to ubiquitination of H4K91 creating a binding site for 53BP1. Interaction of BBAP with PARP9 leads to mono(ADP-ribosyl)ation of ubiquitin moiety preventing elongation of ubiquitin chain. **(B)** Crosstalk between acetylation and ubiquitination. TIP60 acetylates H2AXK5 creating a recognition site for UBC13, which then polyubiquitinates H2AXK119 resulting in eviction of H2AX. H2AK15 can be acetylated or ubiquitinated. When acetylated by TIP60, it is recognized by BRCA1 but prevents the action of RNF8/RNF168, thus promoting HR. When ubiquitinated by RNF8/RNF168, it is recognized by 53BP1 promoting NHEJ. Acetylation of the ubiquitin moiety similarly to mono(ADP-ribosyl)ation prevents elongation of ubiquitin chain. P, phosphorylation, Ac, acetylation, Ub, ubiquitin.

Poly(ADP-ribosyl)ation has extensive crosstalk with other PTM types in DDR, including ubiquitination. CHFR is one of the E3 ubiquitin ligases recruited to DSBs by direct interaction between its zinc finger domain and PAR chains (Figure 5A) (Oberoi et al., 2010). Interestingly, CHFR shares structural similarities with RNF8, with the exception that RNF8 is recruited to lesions in a phosphorylation-dependent manner. At the sites of DSBs, CHFR ubiquitinates auto-PARylated PARP1 with the K48- and K63-linked ubiquitin chains and promotes PARP1 degradation (Liu et al., 2013). It is thought that CHFR can bind and ubiquitinate PARylated histones as well, however, direct evidence is missing. Ubiquitination of PARP1 by CHFR leads to extraction of PARP1 from chromatin and spatiotemporal restriction of PAR spreading, thus controlling the early stage of damage response in a negative feedback loop. CHFR, therefore, belongs to the early-response ubiquitin modifiers in DDR. The E3 ligase TRIP12 can recognize the PAR chains on PARP1 and promote its removal from chromatin as well (Gatti et al., 2020). Another E3 ubiquitin ligase promoting proteasomal degradation of repair proteins and PARP1 in a PAR-dependent manner is RNF146 (Figure 5A) (Kang et al., 2011). Interestingly, RNF146 requires PARylation not only for interaction with its targets but also for ubiquitin ligase activity (DaRosa et al., 2015). The RNF146 RING domain responsible for ubiquitination requires non-covalent binding to PAR to undergo conformational change and switch from an inactive to an active state. Therefore, PARylation serves both as an upstream signal to ubiquitination and as a physical activator of ubiquitin modifiers.

The master regulator of DNA repair BRCA1 was reported to recruit to the sites of DSBs in a PAR-dependent manner as well (Li et al., 2013; Li and Yu, 2013; Hu et al., 2014). It requires the BRCT domain of the BRCA1 interaction partner BARD1 to bind PAR and initiate the homologous recombination pathway. This example of the poly(ADP-ribosyl)ation and ubiquitination crosstalk and its importance for cell survival provides a strategy for cancer therapies.

The F-box containing ubiquitin modifiers were observed to have crosstalk with poly(ADP-ribosyl)ation as well. The mammalian FBXL10-RNF68-RING1B ubiquitin ligase complex (FRRUC) is known as part of the non-canonical PRC1.1 which monoubiquitinates H2A histones on K119 to initiate transcriptional repression in undamaged cells (Wang et al., 2004; Wu et al., 2013). The FRRUC complex is rapidly recruited to the sites of DNA damage in a PARP1- and TIMELESS-dependent manner, however, the exact mechanism is still unknown (Rona et al., 2018). When at the DSBs, the FRRUC non-canonical PRC1 complex facilitates recruitment of the canonical PRC1 complexes containing the E3 ligases BMI1 and MEL18 and mediates H2AK119 ubiquitination. The FRRUC complex was shown to be required for the transcriptional repression, the H2A/H2A.Z histone exchange, and HR damage signaling. Interestingly, depletion of FRRUC impaired the K63-linked ubiquitination by the RNF8/RNF168 axis, but not γH2AX or MDC1 foci formation (Rona et al., 2018). Another F-box E3 ligase that binds PAR chains at double-strand breaks was already mentioned in the previous chapter. FBXW7 is a factor in the NHEJ pathway and is recruited to the sites of DSBs in the ATM-mediated manner. How exactly was not clear until it was discovered that the WD40 domain of FBXW7 binds to PARP1-produced PAR chains (Zhang et al., 2019). Thus, the rapid recruitment of FBXW7 to PAR on damaged chromatin promotes ATM-dependent phosphorylation and retention at damage sites, XRCC4 ubiquitination, and activation of NHEJ. Which PARylated targets are exactly recognized by the FBXW7 reader domain is yet to be discovered, since PAR chains occur not only on histones but on PARP1, PARP2, and repair proteins as well. It is important to mention, that due to almost immediate poly(ADP-ribosyl)ation at the DSB sites, its readers are recruited rapidly too.

The BBAP E3 ligase (also known as DTX3L) was shown to selectively ubiquitinate histone H4 and indirectly promote 53BP1 recruitment to DSBs (Yan et al., 2009). Together with its partner, the macrodomain-containing protein BAL1, BBAP forms a complex that specifically binds PARP1-mediated PAR chains (Figure 5A). Interestingly, it is the BAL1 protein that recognizes PAR and BBAP is tethered to the sites of DNA damage *via* its interaction with BAL1 (Yan et al., 2013). Only together with BAL1 BBAP ligase is able to recruit to DSB and initiate the early ubiquitination wave, which ensures correct damage signaling and repair. The study also revealed that the PARP-BAL1-BBAP ubiquitination axis is functionally independent and nonredundant from the ATM-MDC1-RNF8 one, and they both significantly impair repair both at early and late timepoints (Yan et al., 2013). Strikingly, 53BP1, RAP80, and BRCA1 recruitment *via* PARP1-mediated BAL1-BBAP ubiquitination and *via* γH2AX-dependent RNF8-induced ubiquitination were shown to be separate mechanisms as well, which hints that the recruitment of the major repair proteins is a multi-regulated process. The BBAP ligase can form a complex with other partners apart from BAL1. Another study showed that BBAP can interact with PARP9, a mono(ADP-ribosyl)transferase, which was reported as being enzymatically inactive (Figure 5A) (Yang et al., 2017). When heterodimerized with BBAP, the BBAP-PARP9 heterodimer complex mediates reversible NAD + -dependent mono(ADP-ribosyl)ation of the ubiquitin moiety on Gly76 which is used for conjugation to substrates. As a result, the ADP-ribosylated Ub can no longer be used for ubiquitination and that restricts the E3 ligase activity of BBAP. It was observed that the BBAP-PARP9 complex is recruited to sites of DNA damage. The function of BBAP in NHEJ is regulated by the NAD + concentration and PAR chains. This, in turn, modulates ubiquitin mono(ADP-ribosyl)ation by PARP9. The authors suggested that under normal conditions the effect of ubiquitin mono(ADP-ribosyl)ation is not necessary to inhibit ubiquitin-conjugation, but rather to suppress the formation of polyubiquitin chains by restricting the reaction to a single round of ubiquitin transfers (Yang et al., 2017).

BBAP E3 ligase belongs to the family of the DELTEX ubiquitin ligases (DTX1 to DTX4 and DTX3L), which contain the RING domain and the conserved Deltex C-terminal (DTC) domain. A recent comprehensive study on the interactome and ubiquitination targets of the DTX2 E3 ligase found a large share of the DDR proteins among them (Ahmed et al., 2020). The DTC domain was reported as a new PAR-binding domain involved in PAR-guided ubiquitination by DTX2. This process is organized similarly to the phosphodegrons but in this case, DTX2 ubiquitinates proteins modified by PARP1/PARP2.

As PAR-chains and intrinsically disordered proteins are involved in the formation of phase-separated domains, there might be more ubiquitin modifiers to modulate this process at the sites of DNA damage.

### Acetylation and Ubiquitination

The acetyltransferase TIP60 has several effects on ubiquitination in DDR. The TRRAP-TIP60 complex together with the ATPase p400 acetylates histones H4, H2AX, and ATM and promotes chromatin relaxation at DSBs (Sun et al., 2005; Murr et al., 2006; Ikura et al., 2007; Xu et al., 2010). This facilitates the binding of RNF8/RNF168 and subsequent ubiquitination in the vicinity of breaks. Activating transcription factor 2 ATF2 and E3 ligase complex Cul3/Roc1 in turn control the stability of TIP60 and, therefore, activity of ATM, which requires to be acetylated upon DNA damage (Bhoumik et al., 2008). The depletion of either component of the TRRAP-TIP60 complex impaired both acetylation and ubiquitination at chromatin and in turn hindered recruitment of 53BP1 and BRCA1. In response to DNA damage, TIP60 acetylates H2AX histone on K5 independently of its phosphorylation (Figure 5B). In turn, acetylated H2AX is required for the UBC13-mediated polyubiquitination of H2AX on K119 (Ikura et al., 2007). This acetylation-guided ubiquitination cascade promotes H2AX release from chromatin, which further relaxes the structure and facilitates RAD51 recruitment. Interestingly, the acetylation of the H2A N-terminal region in pre-existing chromatin was found to directly impair subsequent RNF20-RNF40-dependent ubiquitination of H2BK120 (Wojcik et al., 2018). This can bring novel insight into how acetylation regulates downstream ubiquitination in DDR.

The competition between acetylation and ubiquitination for the same residues was shown to regulate pathway choice and chromatin structure at the damage sites. H2AK15 acetylated by TIP60 blocks the ubiquitination and therefore inhibits 53BP1 recruitment promoting HR (Figure 5B) (Jacquet et al., 2016). Another site that can be both exclusively acetylated and ubiquitinated is H2BK120. The levels of acetylation were reported to increase in response to DSBs and the levels of ubiquitinated H2BK120 decreased (Clouaire et al., 2018). As both modifications were shown to alter the higher-order compaction state of the chromatin fiber (Fierz et al., 2011), it is clear that the acetylation-ubiquitination switch is an important part of chromatin remodeling at the sites of the breaks.

The ubiquitin modifiers can be tethered to DSBs by binding acetylated histone marks. The bromodomain-containing E3 ligase TRIM66 was observed to bind unmethylated H3R2-H3K4 and H3K56ac in a combinational manner at the damage sites (Chen et al., 2019). In wild-type cells, H3K56ac is rapidly deacetylated by SIRT6, HDAC1, and HDAC2 at the very early stage of DDR, but the knockdown of TRIM66 causes retention of this mark and impairs repair (Tjeertes et al., 2009; Miller et al., 2010; Battu et al., 2011; Toiber et al., 2013). TRIM66 was found to localize to DSBs in an H3K56ac-dependent manner and subsequently recruit SIRT6 to induce deacetylation, however, it is unknown if this regulatory circuit involves ubiquitination activity of TRIM66.

Moreover, the ubiquitin moiety can be acetylated on either K6 or K48 which does not affect the monoubiquitination but inhibits the formation of K11-, K48-, and K63-linked polyubiquitin chains (Ohtake et al., 2015). Interestingly, one of the targets getting modified with acetylated ubiquitin is histone H3 (Figure 5B). Another study analyzed the effect of acetylated ubiquitin on the catalytic activity of the E1 enzyme UBA1. It was observed that acetylation on all seven internal lysines can impair the conformational change required for the E1-E2 transfer and Ub-conjugation to the E2 enzyme, thus resulting in impaired target ubiquitination (Lacoursiere and Shaw, 2021). The role of acetylated ubiquitin in DDR signaling is yet to be investigated.

### SUMOylation and Ubiquitination

**FIGURE 6:**
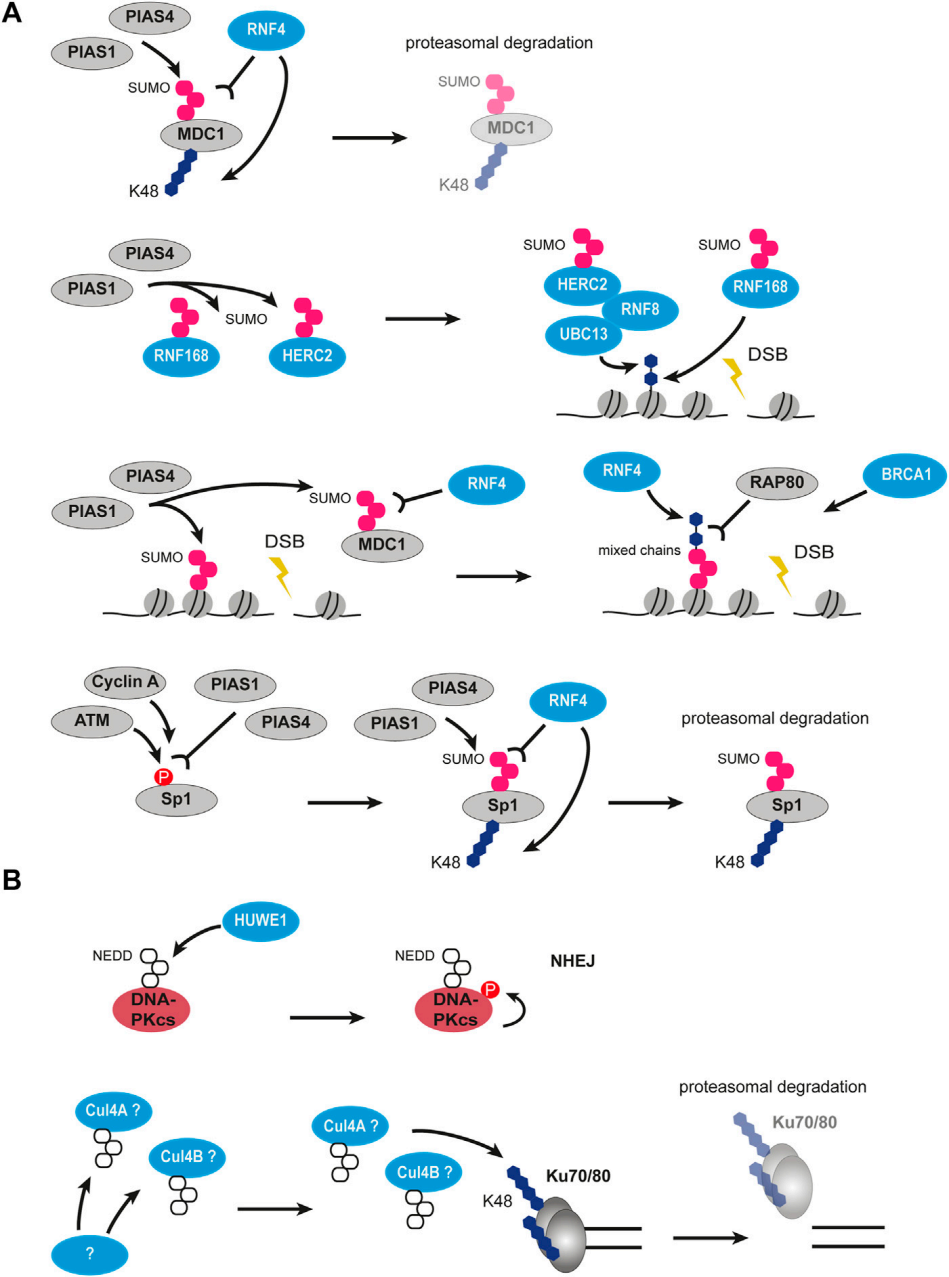
Crosstalk between SUMOylation and ubiquitination, as well as NEDDylation and ubiquitination in DSB signaling and repair. **(A)** Crosstalk between SUMOylation and ubiquitination. MDC1 can be SUMOylated by PIAS1 and PIAS4 and then recognized by RNF4 leading to its polyubiquitination and degradation. PIAS1 and PIAS4 can also SUMOylate RNF168 and HERC2 leading to their activation. Activated HERC2 interacts with RNF8 and UBC13 and ubiquitinates histones together with activated RNF168. PIAS1 and PIAS4-mediated SUMOylation of MDC1 and histones recruits RNF4, which builds mixed SUMO-ubiquitin chains recognized by RAP80. Phosphorylated Sp1 is recognized PIAS1 and PIAS4 leading to its SUMOylation. SUMOylated Sp1 is recognized by RNF4 and gets polyubiquitinated and degraded. **(B)** Crosstalk between NEDDylation and ubiquitination. HUWE1 ubiquitin ligase can NEDDylate DNA-PKcs leading to its autophosphorylation and promoting NHEJ. NEDDylation of potentially cullins leads to polyubiquitination of Ku70/80 and their degradation. The enzyme performing NEDDylation is unknown. P, phosphorylation; SUMO, SUMOylation; NEDD8, NEDDylation; U, ubiquitin.

The addition of SUMO (small ubiquitin-related modifier) is also involved in DDR. There are three functionally redundant SUMO proteins encoded in mammalian cells, SUMO1, SUMO2, and SUMO3. However very similar to ubiquitination, SUMOylation uses its own enzymatic system consisting of an E1 activating enzyme (SAE1/SAE2), an E2 ligase (UBC9 also known as UBE2I), and various E3 ligases (Celen and Sahin, 2020). Ubiquitination has tight crosstalk with SUMOylation, the cascades are various in their directionality and organization. SUMO proteins recruit to IRIF 4 h after damage (Galanty et al., 2009). The recruitment occurs in a PIAS1- and PIAS4-dependent manner, two E3 ligases of the SUMO system. It was found that the RNF8/RNF168-mediated ubiquitination on chromatin is required for the SUMO signaling at DSBs and the SUMOylation is functionally divided into 53BP1-SUMO1 and BRCA1-SUMO2/3 pathways (Figure 6A). So far SUMOylation was reported for multiple repair proteins, including MDC1, 53BP1, BRCA1, RPA, CtIP, RNF8, and HERC2 (Galanty et al., 2009, 2012; Morris et al., 2009; Danielsen et al., 2012; Luo et al., 2012; Yin et al., 2012; Locke et al., 2021). Many DDR ubiquitin modifiers require being SUMOylated for their E3 ligase activity, e.g. BRCA1/BARD1 heterodimer. Interestingly, SUMO signaling not only relies on RNF8 and K63-linked ubiquitination on chromatin but also participates in ubiquitination stability and spreading (Danielsen et al., 2012).

One of the essential SUMO cascades at the sites of DNA damage is mediated by the SUMO-targeted ubiquitin ligase (STUbL) RNF4. RNF4 is recruited to DSBs in a PIAS1-, PIAS4-dependent manner and requires its SUMO interaction motifs (Galanty et al., 2012). When bound to SUMOylated proteins like MDC1, RNF4 ubiquitinates them and targets them for proteasomal degradation thus ensuring the correct timing of DDR steps (Figure 6A). The lack of RNF4 results in severe DNA repair defects and constant phosphorylation of H2AX histone as a signal of persistent damage (Luo et al., 2012; Yin et al., 2012). Additionally, deSUMOylation by the deSUMOylase SENP2 provides a further pathway specific switch. It was shown that the extraction of MDC1 after RNF4 mediated ubiquitination and simultaneous SUMOylation is blocked by the SENP2 mediated deSUMOylation and thus promotes NHEJ (Garvin et al., 2019). Aside from repair proteins removal, RNF4 was proposed to facilitate ubiquitination signaling at the DSBs. The RAP80 protein is an important mediator of BRCA1 recruitment to damaged chromatin and requires both SUMO and ubiquitin modifications (Guzzo et al., 2012; Hu et al., 2012). RNF4 is thought to produce the hybrid SUMO-ubiquitin chains, which tether RAP80 and subsequently BRCA1 promoting HR (Figure 6A). The crucial role of SUMOylation and ubiquitination crosstalk in DNA repair pathway choice is getting more evidence with time. A recent study identified Sp1 as a target for SUMO-dependent RNF4 ubiquitination (Swift and Azizkhan-Clifford, 2022). The cascade involves DNA damage-induced phosphorylation of Sp1 by ATM and Cyclin A/CDK2 upon entry in the S phase, which is necessary for subsequent SUMOylation of Sp1 on K16 (Figure 6A). The serial events of phosphorylation and SUMOylation on Sp1 are recognized by RNF4 which ubiquitinates Sp1 and initiates its degradation. As Sp1 affects the localization of 53BP1, its degradation results in the removal of both Sp1 and 53BP1 from DSBs. The search for targets of RNF4 is ongoing and the number of players identified in SUMOylation-ubiquitination crosstalk is growing (Kumar et al., 2017).

### Neddylation and Ubiquitination

NEDD8 (neural precursor cell expressed developmentally downregulated 8) is another ubiquitin-like protein that shares the highest similarity to ubiquitin, it is ∼60% identical and ∼80% homologous to ubiquitin (Jentsch and Pyrowolakis, 2000). However, as SUMOylation, the neddylation has its own enzymatic system in tight coordination with ubiquitination, and some ubiquitin ligases were shown to be able to perform neddylation due to the high similarity between NEDD8 and ubiquitin. The modification with NEDD8 was identified as the second most abundant ubiquitin-like modification following SUMO that accumulates at DSBs (Ma et al., 2013). At the DSBs, this NEDD8 accumulation was observed to be mediated by the E3 ubiquitin ligase RNF111 on the histone H4 N-terminus. Interestingly, H4 polyneddylation can be recognized by the MIU domain of RNF168, thus providing another regulating node for the RNF8/RNF168 ubiquitination axis (Ma et al., 2013).

Neddylation-guided ubiquitination at the sites of DNA damage regulates repair machinery and repair pathway choice as well. Ku70/80 was reported to be ubiquitinated on K195, K265, and K481 in Ku80 and K114 in Ku70 in a neddylation-dependent manner by a yet unknown factor (Figure 6B) (Brown et al., 2015). The ubiquitination of Ku70/80 causes its disassembly from the broken ends and therefore can promote resection-dependent repair. Consistently with the fact that cullins are the predominant targets of neddylation, the authors observed CUL4A and CUL4B as neddylation-dependent interacting partners of Ku70/80.

The dynamic regulation of neddylation and deneddylation at the DSBs was confirmed to guide repair pathway choice by controlling the length of CtIP-resected DNA (Jimeno et al., 2015). The authors reported that the RNF111/UBE2M-dependent neddylation inhibits resection and promotes NHEJ, while the deneddylation is required to initiate HR. At the sites of DNA damage, CtIP and its partner BRCA1 constitutively interact with neddylated proteins, therefore the balance between neddylation and its removal is crucial for the correct repair choice. Interestingly, the neddylation level on damaged chromatin regulates not only NHEJ/HR choice but also the fine balance between various variants of HR (Jimeno et al., 2015).

As a further example, DNA-PKcs was shown to be polyneddylated at its kinase domain by the E3 ubiquitin ligase HUWE1 (Figure 6B) (Guo et al., 2020). This modification is necessary for the autophosphorylation of DNA-PKcs on Ser2056 and efficient NHEJ.

There are more ubiquitin-like modifications, which have been described recently such as UFMylation, FATylation and ISGylation but little is known about their roles in DSB signaling and repair.

## Ubiquitination, DNA Damage and Disease

Due to the abundance and versatility of the ubiquitin network, the dysregulation of any of its components can potentially lead to pathogenesis. Ubiquitination defects were reported to cause cancer, neurodegenerative diseases, immune pathologies, and muscle atrophy-related diseases (Popovic et al., 2014; Zheng et al., 2016). Since the first successful clinical application of proteasomal inhibitors (Hideshima et al., 2001), the search for a druggable ubiquitinome never stopped (Table 3).

**TABLE 3:** Inhibitors of ubiquitin modifiers.

Target	Name	References
VCP	NMS-873	Magnaghi et al. (2013)
p97	CB-5083	Kilgas et al. (2021)
MDM2	Nutlin	Vassilev et al. (2004)
MDM2	APG-115	Rasco et al. (2019a)
MDM2	CGM097	Holzer et al. (2015)
E3 ligases	PROTACs	Bondeson et al. (2015)
TRIM24	dTRIM24	Gechijian et al. (2018)
Cullin-RING ligases	MLN4924	Zhao et al. (2014); Tong et al. (2017)
KEAP1	Omaveloxolone	Lynch et al. (2019)
Proteasome components	PS-341	Teicher et al. (1999)
Proteasome components	Marizomib	Potts et al. (2011)
Proteasome components	MLN9708	Kupperman et al. (2010)
Proteasome components	NPI-0052	Chauhan et al. (2005)
Proteasome components	CEP-18770	Piva et al. (2008)
E3 ligases	CC-122	Rasco et al. (2019b)
E3 ligases	CC-220	Bjorklund et al. (2020)
SMURF1	HS-152	Tian et al. (2019)
DUBs	VLX1570	Wang et al. (2015)

The ubiquitination defects in DNA damage and repair are mostly related to genome instability. A well-known example are germline mutations in the BRCA1 gene, often in the RING domain region, that predispose to breast and ovarian cancer (Maxwell and Domchek, 2012). BRCA1 recruitment is mediated by the RNF8/RNF168 ubiquitination signaling and its dysregulation leads to severe consequences. Mutations inactivating the RNF168 gene were shown to cause the RIDDLE syndrome, a rare disease characterized by radiosensitivity, immunodeficiency, dysmorphic features, pulmonary failure, and learning disabilities (Stewart et al., 2009, 2007). The RNF168 can harbor various mutations and therefore result in different symptoms. A nonsense mutation leading to the loss of both ubiquitin-binding domains MIU1 and MIU2 was described to cause ataxia, growth retardation, microcephaly, immunodeficiency, and radiosensitivity (Devgan et al., 2011). Interestingly, the disease phenotype was accompanied by persistent chronic inflammation from unrepaired DNA damage that caused the idiopathic pulmonary fibrosis. RNF168 mutated in MIU1 and 2 was not able to retain 53BP1 and BRCA1 at the sites of the damage, thus impairing the RNF8-RNF168-HERC2-BRCA1 chromatin ubiquitin ligase cascade in DDR (Devgan et al., 2011). The paramount role of the ubiquitin ligases in maintaining genome stability is highlighted in the Fanconi anemia disease. Fanconi anemia is a rare disorder that results in bone marrow failure, cancer predisposition, and genomic instability. Fanconi anemia patients suffer chromosome fragility and hypersensitivity to drugs that induce DNA interstrand crosslinks. Often patients develop solid tumors such as squamous cell carcinomas in their 20s (Pollard and Gatti, 2009). There are more than 20 genes identified in the Fanconi anemia (FA) pathway that assemble in multiple complexes in the FA–BRCA DNA-damage response network (Wang, 2007; Fang et al., 2020). Eight of them (FANCA, B, C, E, F, G, L, and M) form the so-called FA core complex together with FANCA-associated polypeptides FAAP100 and FAAP24, a nuclear E3 ubiquitin ligase complex that monoubiquitinates the FANCD2/FANCI heterodimer (FA-ID complex) in response to damage. This results in the stabilization of the FA-ID complex on DNA and subsequent interaction with BRCA2, PALB2, RAD51, and BRIP1 to promote homologous recombination (Garcia-Higuera et al., 2001; Alcón et al., 2020). Most FA cases harbor mutations leading to a ubiquitination defect (Tan and Deans, 2017). The FA-ID ubiquitination is required for prevention of bone marrow failure, and FA patients with FANCD2 mutations were reported to have an early onset of bone marrow failure (Boisvert and Howlett, 2014; Tan et al., 2020). Depletion of any FA proteins responsible for the ubiquitination sensitizes cisplatin-resistant human lung adenocarcinoma cells to cisplatin treatment (Chen et al., 2016). Mutations in the FA genes deregulate the DDR and the predisposition to the BRCA1-and BRCA2-dependent cancers (Kais et al., 2016).

Various combinations of mutations in genes coding for different ubiquitin modifiers result in various pathological phenotypes. The genetic deletion of RNF8 and CHFR sensitizes mice to ionizing radiation and results in the development of T-cell lymphoma, emphasizing the importance of the combined action of these phospho- and PAR-targeted ubiquitin ligases in the DDR (Wu J. et al., 2011). The RNF8 knockout in mice causes growth retardation, sensitivity to ionizing radiation, impaired spermatogenesis, and defective immunoglobulin class-switch-recombination, similar as RNF168 and 53BP1 knockouts (Manis et al., 2004; Li et al., 2010; Santos et al., 2010). Interestingly, similar effects as for RNF8 and RNF168 knock out were observed for RNF4 deficiency, which links SUMOylation to ubiquitination cascades in DDR (Vyas et al., 2013).

The susceptibility of the ubiquitination cascades involved in DDR are not only the source of genetic instability in normal cells but can be also used to sensitize cancer cells to ionizing radiation or chemotherapy. A well-known example is PARP inhibition, which leads to impaired homologous recombination in cells with BRCA1 and BRCA2 mutations (Bryant et al., 2005). The VCP/p97 complex mediates the turnover of K48-linked ubiquitin-labeled proteins at the sites of DSBs thus facilitating timely repair (Meerang et al., 2011). The VCP/p97 inhibitor NMS-873 was shown to induce unfolded protein response, autophagy, and cell death (Magnaghi et al., 2013). Additionally, VCP/p97 inhibition by the specific small-molecule inhibitor CB-5083 leads to cell death after IR due to the excessive MRN-mediated resection (Kilgas et al., 2021). Many tumors overexpress MDM2 (human analog HDM2), the E3 ubiquitin ligase that ubiquitinates p53 and negatively controls its levels thus promoting cell survival (Ladanyi et al., 1993). Therefore, the inhibitors of MDM2 are promising anti-cancer drugs. One of the most potent MDM2 small-molecule inhibitors identified in high-throughput screening is the Nutlin family, analogs of cis-imidazoline (Vassilev et al., 2004). Currently two MDM2 inhibitors are in clinical trials: APG-115 is in Phase I, and CGM097 in Phase II (Holzer et al., 2015; Rasco et al., 2019a; Rasco et al., 2019b). Inhibiting MDM2 activity, the inhibitors restore the anti-cancer activity of p53 and lead to apoptosis. The MLN4924 inhibitor blocks Cullins NEDDylation and inactivates Cullin-RING ligases which, in turn, triggers cell cycle arrest, apoptosis, senescence, and autophagy in many cancer cells (Zhao et al., 2014; Tong et al., 2017). In addition, it was reported that the accumulation of substrates of the Cullin-RING ligases (p21, p27, Wee1), trigger DNA damage, and induce cell cycle arrest at the G2/M stage (Tong et al., 2017).

The development of new small-molecule inhibitors targeting ubiquitin modifiers in the DDR pathways for the treatment of cancer and other diseases is underway and requires the expansion of our knowledge about the ubiquitination network.

## Conclusion

Although plenty of novel ubiquitin modifiers controlling DDR pathways were discovered in recent years, there is still missing knowledge. Due to the immense complexity of the ubiquitin-dependent signaling system, DNA damage signaling and repair are precisely spatiotemporally regulated in the chromatin context. Recent studies unveiled novel functions of the ubiquitin cascades at the DSBs, such as phase separation, chromatin mobility, DSB clustering, and homology search. An additional layer of regulation, the phosphorylation of ubiquitin, was recently reported for its role in DDR. In the coming years, many novel ubiquitin modifiers and ubiquitination cascades are expected to be discovered and more emphasis will be placed on the crosstalk between ubiquitination and other PTMs taking place at the damage sites. At last, the knowledge we gain about the ubiquitination system in DDR would give us a potent tool to develop therapeutic approaches for cancer and other diseases.

## Glossary

ATM: ataxia-telangiectasia mutated
ATR: ATM and RAD3-related
CDK: cell cycle-dependent kinase
CFS: common fragile sites
cNHEJ: canonical NHEJ
DDR: DNA damage response
DNA-PKcs: DNA-dependent protein kinase catalytic subunit
DSB: double-strand break
DTC: Deltex C-terminal domain
DUB: deubiquitinase
ERK: extracellular signal-regulated kinase
FA: Fanconi anemia
FRRUC: FBXL10-RNF68-BMI1 ubiquitin ligase complex
HDAC: histone deacetylase
HR: homologous recombination
IR: ionizing radiation
IRIF: irradiation-induced focus
LET: linear energy transfer
LLPS: liquid-liquid phase separation
MIU: motif interacting with ubiquitin
NEDD8: neural precursor cell expressed developmentally downregulated 8
NER: nucleotide excision repair
NHEJ: non-homologous end joining
PAR: poly(ADP-ribose)
PARP: poly (ADP-ribose) polymerase
PI3: phosphoinositide 3
PI3KK: phosphoinositide 3 kinase-related protein kinase
polyUb: polyubiquitin
PTM: post-translational modification
pUb: phosphoubiquitin
RIDDLE: Radiosensitivity, ImmunoDeficiency Dysmorphic features and LEarning difficulties syndrom
ssDNA: single-strand DNA
STUbL: SUMO-targeted ubiquitin ligase
SUMO: small ubiquitin-like modifier
TAD: topologically associated domain
TSA: Trichostatin A
Ub: ubiquitin
UBD: ubiquitin-binding domain
UBZ: ubiquitin-binding zinc-finger
UIM: ubiquitin-interacting motif
